# Circumtropical distribution and cryptic species of the meiofaunal enteropneust *Meioglossus* (Harrimaniidae, Hemichordata)

**DOI:** 10.1038/s41598-024-57591-0

**Published:** 2024-04-23

**Authors:** Éloïse Defourneaux, Maria Herranz, Maickel Armenteros, Martin V. Sørensen, Jon L. Norenburg, Taeseo Park, Katrine Worsaae

**Affiliations:** 1 Marine Biological Section, Department of Biology, University of Copenhagen, Universitetsparken 4, DK-2100 Copenhagen, Denmark; 2 Area of Biodiversity and Conservation, Superior School of Experimental Science and Technology (ESCET), Rey Juan Carlos University, C/ Tulipán S/N, 28933 Mostoles, Madrid, Spain; 3 Unidad Académica Mazatlán, Instituto de Ciencias del Mar y Limnología, Universidad Nacional Autónoma de México, Av. Joel Montes Camarena S/N, 82040 Mazatlán, México; 4 Natural History Museum Denmark, University of Copenhagen, Universitetsparken 15, DK-2100 Copenhagen, Denmark; 5 Smithsonian National Museum of Natural History, Washington, DC USA; 6 Species Diversity Research Division, National Institute of Biological Resources, Hwangyeong-Ro 42, Incheon, 22689 South Korea

**Keywords:** Phylogenetics, Population genetics, Taxonomy, Marine biology, Biodiversity

## Abstract

Hemichordata has always played a central role in evolutionary studies of Chordata due to their close phylogenetic affinity and shared morphological characteristics. Hemichordates had no meiofaunal representatives until the surprising discovery of a microscopic, paedomorphic enteropneust *Meioglossus psammophilus* (Harrimaniidae, Hemichordata) from the Caribbean in 2012. No additional species have been described since, questioning the broader distribution and significance of this genus. However, being less than a millimeter long and superficially resembling an early juvenile acorn worm, *Meioglossus* may easily be overlooked in both macrofauna and meiofauna surveys. We here present the discovery of 11 additional populations of *Meioglossus* from shallow subtropical and tropical coralline sands of the Caribbean Sea, Red Sea, Indian Ocean, and East China Sea. These geographically separated populations show identical morphology but differ genetically. Our phylogenetic reconstructions include four gene markers and support the monophyly of *Meioglossus*. Species delineation analyses revealed eight new cryptic species, which we herein describe using DNA taxonomy. This study reveals a broad circumtropical distribution, supporting the validity and ecological importance of this enigmatic meiobenthic genus. The high cryptic diversity and apparent morphological stasis of *Meioglossus* may exemplify a potentially common evolutionary ‘dead-end’ scenario, where groups with highly miniaturized and simplified body plan lose their ability to diversify morphologically.

## Introduction

Studies on hemichordate evolution can provide valuable insights into the early evolution of deuterostomes and chordates^1–4^. The marine phylum Hemichordata is divided into two major classes; the solitary and free-living Enteropneusta (acorn worms), and the mostly colonial and sessile tube dwelling Pterobranchia. Gathering about 110 species^5^, the solitary acorn worms are divided into four families: Ptychoderidae, Spengelidae, Harrimaniidae, and Torquaratoridae^6–10^.

The microscopic enteropneust *Meioglossus psammophilus* Worsaae, Sterrer, Kaul-Strehlow, Hay-Schmidt & Giribet, 2012^11^ is the only meiofaunal, solitary adult representative of hemichordates. It presents the usual tripartite body plan of Hemichordata including a proboscis, a collar, and a trunk^12^. Based on shared morphological traits paired with DNA investigations using 18S sequencing, this meiofaunal enteropneust was positioned in Harrimaniidae^11^. In contrast to the typical morphology of this family, *Meioglossus* has a simpler body plan, rather resembling a harrimaniid early juvenile with its small size (less than 700 µm), a short post-anal tail, complete ciliation, single pair of gill pores, and lack of neck skeleton. This, combined with its phylogenetic affinity to Harrimaniidae, suggests that *Meioglossus* is a miniaturized, paedomorphic hemichordate^11^.

Although *Meioglossus* has a larval or early juvenile appearance, its adulthood is supported by the presence of sperm and asexual reproduction by paratomy. However, since no female has yet been found, the larval-looking appearance of the males, raise the questions whether *Meioglossus* i) is truly meiofaunal, or males are just dwarf males of an unknown macroscopic female, or ii) may only exhibit asexual reproduction. Asexual reproduction is found in both enteropneusts and pterobranchs^13–15^, but *Meioglossus* is the only known hemichordate to divide by paratomy^11^. With the absence of females, eggs, and larvae, *Meioglossus* may thus rely entirely on asexual reproduction through paratomy.

*Meioglossus* development is likely direct due to their microscopic size, interstitial lifestyle, and affinity to the direct developing Harrimaniidae^11^. Similar to most benthic meiofauna lacking a pelagic larval stage, the dispersal ability of *Meioglossus* is expected to be limited (yet, see e.g.,^16^). Until now *Meioglossus* is monotypic, only represented by *M. psammophilus* from a few localities in the Caribbean. This so far limited diversity could easily be explained by the so-called taxonomic impediment—the gap between taxonomist expertise and a massive number of species to identify and describe^17^. Moreover, new findings of *Meioglossus*, from disparate geographical Caribbean locations to the East China Sea^18,19^ (Worsaae unpublished) translate the need of population genetic investigations.

Soft-bodied meiofauna may often lack distinctive features and suffer from a ‘meiofaunal syndrome’ i.e. having a small, uniform, elongated and simple worm-like body shape^20,21^. This has led to discussions on morphological stasis or morpho-evolutionary ‘dead-end’, in particular for paedomorphic animals, which have undergone extreme miniaturization and potentially descended from a larval or juvenile ancestor^16,22–24^. Superficial examination at low magnification in the field is insufficient to identify meiofauna species, which generally require thorough morphological examinations. Even though detailed morphological studies and taxonomic expertise is still the most common way of identifying and describing new meiofauna taxa^23,25–27^, morphological methods may not always comply well to these miniature soft-bodied animals^28,29^. *M. psammophilus* was described based on morphologically similar specimens from distant locations of Belize and Bermuda in the Caribbean. Even advanced morphological studies including both Confocal Laser Scanning Microscopy (CLSM) and Transmission Electron Microscopy (TEM) of e.g., nerves, muscles, ciliation, and sperm did not reveal any morphological differences between these disjunct populations. Unfortunately, genetic information was only obtained from one population in Bermuda^11^. New findings of morphologically similar but geographically vastly distant populations of *Meioglossus* casts doubts on the systematic importance of morphological traits for species diagnoses.

Describing species solely based on morphological traits is no longer sufficient. The integration of additional methods, especially molecular sequencing, has become crucial^30^. This has given rise to the field of integrative taxonomy, widely used in various studies^27,31–34^. Integrative taxonomy has been occasionally conflated with another emerging approach known as turbo taxonomy that is characterized by the rapid description of numerous species in ‘fast papers’. Again, a combination of various tools and approaches are used, but it often involves quick assessments of morphological features, while being heavily reliant on molecular data to differentiate new species^35–38^. Molecular-based techniques for species identification is now a firmly established method to comply meiofaunal diversity and beyond^39,40^. DNA taxonomy comprises several approaches (e.g. multi-gene barcoding and combined molecular species delineation approaches) and can generate valuable diagnostic characters to identify molecular distinct but morphologically cryptic species.

During the last decade we discovered and sampled 11 new populations of *Meioglossus*, from field observations all fitting the morphological diagnosis of *M. psammophilus.* The 11 locations were found in tropical and subtropical regions of the West Atlantic and the Indo-Pacific oceans (Cuba, Turks and Caicos Islands, Curaçao, Israel, the Maldives, and South Korea), in addition to the described populations of Belize and Bermuda. We sequenced four genetic markers (16S rRNA, 18S rRNA, COI and H3) from representatives of all 14 populations to address their phylogenetic relationships and species delineation. In addition to morphological field observations, detailed observations on preserved material were conducted searching for distinct morphological differences from the diagnosis of *M. psammophilus*. Based on these analyses, we here describe eight new cryptic species of *Meioglossus* using DNA taxonomy. Biogeographical patterns and possible evolutionary scenarios of *Meioglossus* are discussed.

## Methods

### Sampling and data gathering

**Figure 1:**
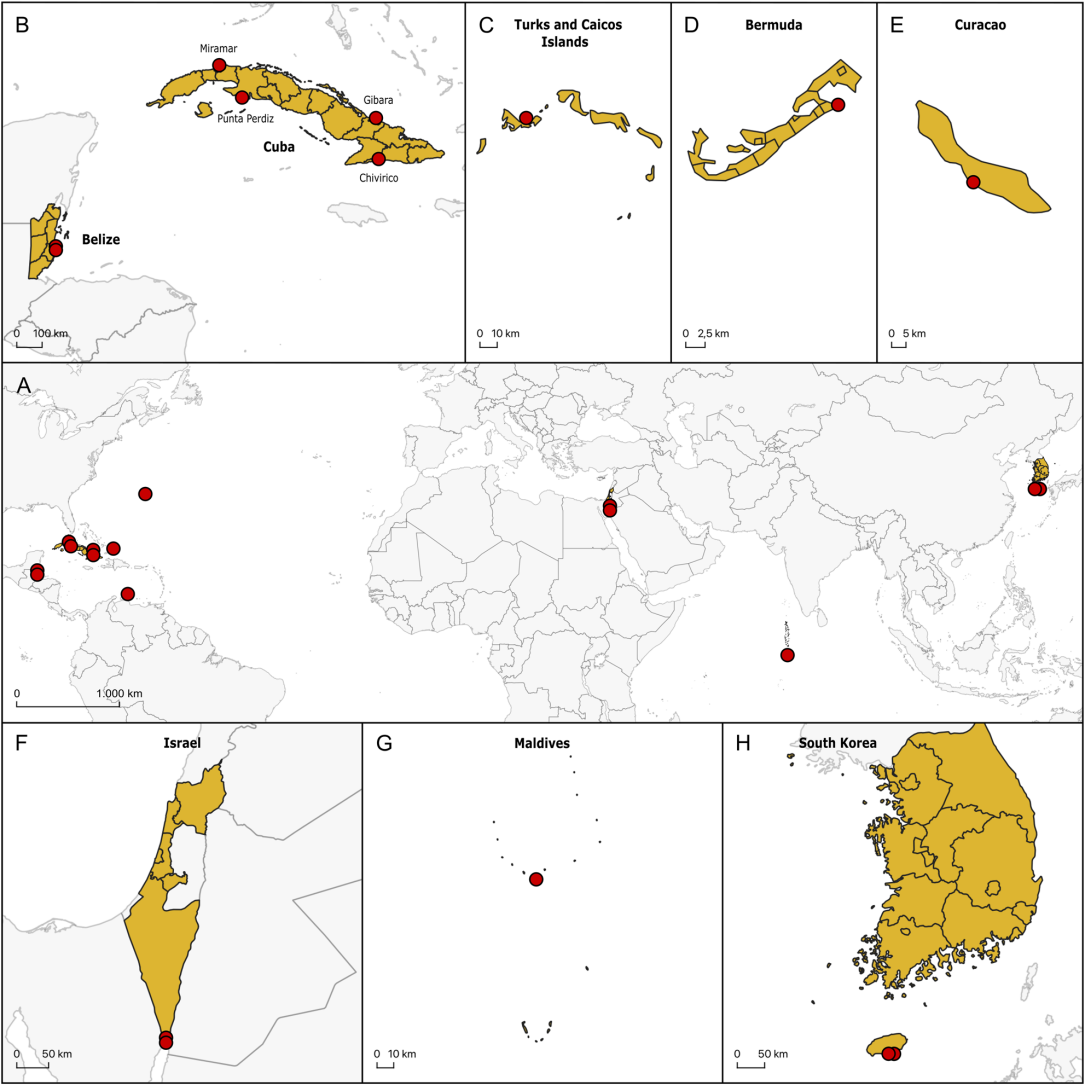
Maps marking the 14 *Meioglossus* sampling localities (red dots). (**A**) World map highlighting all the localities. (**B**) Zoom on Belize and Cuba. (**C**) Zoom on Turks and Caicos Islands. (**D**) Zoom on Bermuda. (**E**) Zoom on Curaçao. (**F**) Zoom on Israel. (**G**) Zoom on the Maldives. (**H**) Zoom on South Korea. Map was generated using QGIS 3.24.2 software (https://www.qgis.org).

*Meioglossus* were sampled over 15 years and multiple expeditions from nine localities in the Caribbean and five localities in the Indo-Pacific (Fig. 1, locality details and collection data are specified in Supplementary Table 1). Fine to coarse coralline sand sediment samples were collected by hand, scuba-diving or dredging, from 0.3 to 33 m depth. Sediment samples were anesthetized using a 1:1 isotonic MgCl_2_ and seawater solution releasing adhesive animals from the sand grains. Samples were then resuspended and animals decantated through a 63-µm cone-shaped mesh^23^. Animals were revitalized in petri dishes with seawater and *Meioglossus* sorted out using a dissecting scope. Specimens were fixed in 2% paraformaldehyde (PFA) or glutaraldehyde (GLU) for morphological analyses or stored in 99% ethanol (EtOH) for molecular analyses.

Type material is deposited in the Natural History Museum of Denmark (NHMD) except for one paratype from Jeju Island deposited in the National Institute of Biological Resources (NIBR).

### Extraction, PCR and sequencing

DNA was extracted from 38 entire specimens (up to five individuals, when possible, of each population), using the Qiagen Dneasy^®^ Blood & Tissue Kit (Cat. no. 69506) following the manufacturer’s instructions. Four common gene markers representing both mitochondrial and nuclear genes, and fast and slow evolving genes, were targeted for resolving the relationships of *Meioglossus*: mitochondrial 16S ribosomal RNA (16S rRNA, 450 base pairs (bp)), Cytochrome *c* oxidase subunit I (COI, 650 bp), nuclear 18S ribosomal RNA (18S rRNA, 1800 bp) and Histone 3 (H3, 340 bp).

Polymerase Chain Reactions (PCR) were performed following a previously optimized protocol for interstitial meiofaunal invertebrates^41,42^ (check Supplementary Table 2 for list of primers, sequences, and references). Amplified PCR products were visualized on 1% agarose gels stained with GelRed® (Biotium, 41003) (Hames, 1998), and purified using the E.Z.N.A.^®^ Cycle Pure Kit, following the manufacturer’s instructions. They were later shipped to Macrogen Europe (The Netherlands, Amsterdam) for sequencing^43^.

### Assembly, alignment, and outgroup selection

Chromatogram visualization and contig assembly were performed using either Sequencher v4.8 (Gene Codes Corporation, Ann Arbor, MI USA) or Geneious prime v2021.2.2 (Dotmatics)^44^. Each consensus sequence was verified for contamination on the NCBI Standard Nucleotide Blast online platform, using the BLAST tool (Basic Local Alignment Search Tool)^45^. All sequences were deposited in GenBank^®^.

Outgroup selection was based on previous hemichordate phylogenetic analyses^9,11,46^ as well as availability of relevant sequences on GenBank. *Saxipendium coronatum* Woodwick & Sensenbaugh, 1985^47^, *Harrimania planktophilus* Cameron, 2002^48^, *Protoglossus koehleri* Caullery & Mesnil, 1900^49^, *Saccoglossus mereschkowskii* (Wagner, 1885)^50^, *Saccoglossus pusillus* Ritter, 1902^51^ and *Saccoglossus kowalevskii* (Agassiz, 1873)^52^ are all representatives of the Harrimaniidae, like *Meioglossus*. Two species of Ptychoderidae; *Balanoglossus carnosus* Müller in Spengel, 1893^53^ and *Balanoglossus clavigerus* Delle Chiaje, 1829^54^ were also included in the analyses (Table 1).

**Table 1:** Molecular specimens used in this presented study, including GenBank accession number.

Species	*Meioglossus* localities	ID extraction	Genbank accession numbers				Publications
			16S rRNA	18S rRNA	COI	H3	
*Balanoglossus carnosus*	–	–	LC120738	LC120751	NC_001887	–	Urata, 2016 Castresana et al. 1998
*Balanoglossus clavigerus*	–	–	EU728425	–	NC_013877	FN908646	Cannon et al. 2009 Perseke et al. 2010
*Saxipendium coronatum*	–	–	EU728423	EU728433	–	–	Cannon et al. 2009
*Saccoglossus mereschkowskii*	–	–	KF683545	KF683588	–	–	Cannon et al. 2013
*Saccoglossus kowalevskii*	–	–	NC_007438	L28054	NC_007438	XM_006825735	Smith et al. 2003 Turbeville et al. 1994
*Saccoglossus pusillus*	–	–	EU728422	AF236800	–	–	Cannon et al. 2009 Cameron et al. 2000
*Harrimania planktophilus*	–	–	EU728421	AF236799	–	–	Cannon et al. 2009 Cameron et al. 2000
*Protoglossus koehleri*	–	–	EU728420	EU728432	–	–	Cannon et al. 2009
***Meioglossus psammophilus***	Cuba—Northwest—Miramar	KW512	**OR831134**	**OR831174**	–	–	This paper
	Cuba—Northwest—Miramar	KW802	**OR831156**	**OR831173**	**OR941456**	**OR908945**	This paper
	Cuba—Southwest—Punta Perdiz	KW511	**OR831133**	**OR831172**	–	**OR908937**	This paper
	Cuba—Southwest—Punta Perdiz	KW546	**OR831136**	OR831195**	**OR941447**	–	This paper
	Belize—East—Carrie Bow Cay st. 9	KW099 A	**OR831128**	OR831167*	–	–	This paper
	Belize—East—Carrie Bow Cay st. 9	KW099 B	**OR831129**	OR831168*	–	–	This paper
	Belize—East—Carrie Bow Cay st. 9	KW099 C	**OR831130**	OR831169*	–	–	This paper
	Belize—East—Carrie Bow Cay st. 9	KW099 D	**OR831131**	OR831170*	–	–	This paper
	Belize—East—Carrie Bow Cay st. 9	KW099 E	**OR831132**	OR831171*	–	–	This paper
***Meioglossus bermudensis* sp. nov**	Bermuda—Northeast—Windsor beach	KWB	JX855287	JF900488	**OR941435**	**OR908946**	Worsaae et al. 2012
***Meioglossus chiviricoensis* sp. nov**	Belize—East—Carrie Bow Cay st. 4	KW096 A	–	OR831163*	–	–	This paper
	Belize—East—Carrie Bow Cay st. 4	KW096 B	**OR831127**	OR831164*	**OR941431**	–	This paper
	Cuba—Southeast—Chivirico	KW702	**OR831138**	–	**OR941432**	–	This paper
	Cuba—Southeast—Chivirico	KW785	**OR831140**	**OR831165**	**OR941433**	**OR908953**	This paper
	Cuba—Southeast—Chivirico	KW786	**OR831141**	**OR831166**	**OR941434**	–	This paper
***Meioglossus curacaoensis* sp. nov**	Curaçao—West—Sint Michiel	KW795	**OR831149**	**OR831178**	**OR941452**	**OR908942**	This paper
	Curaçao—West—Sint Michiel	KW796	**OR831150**	**OR831179**	–	**OR908943**	This paper
	Curaçao—West—Sint Michiel	KW797	**OR831151**	**OR831180**	**OR941453**	**OR908944**	This paper
	Curaçao—West—Sint Michiel	KW798	**OR831152**	–	**OR941454**	–	This paper
	Curaçao—West—Sint Michiel	KW799	**OR831153**	–	**OR941455**	–	This paper
***Meioglossus eilatensis* sp. nov**	Israel—South—Eilat st. 16	KW800	**OR831154**	**OR831193**	–	**OR908951**	This paper
	Israel—South—Eilat st. 16	KW801	**OR831155**	OR831194*	**OR941457**	–	This paper
***Meioglossus iuiensis* sp. nov**	Israel—South—Eilat st. 32	KW513	**OR831135**	**OR831181**	**OR941436**	–	This paper
	Israel—South—Eilat st. 32	KW790	**OR831144**	**OR831183**	**OR941438**	**OR908952**	This paper
	Israel—South—Eilat st. 32	KW791	**OR831145**	**OR831184**	**OR941437**	–	This paper
***Meioglossus jejuensis* sp. nov**	South Korea—South—Jeju st. 19	KW718	**OR831139**	OR831188*	**OR941442**	–	This paper
	South Korea—South—Jeju st. 19	KW788	**OR831142**	OR831189*	**OR941443**	**OR908949**	This paper
	South Korea—South—Jeju st. 19	KW789	**OR831143**	**OR831190**	**OR941444**	**OR908947**	This paper
	South Korea—South—Jeju st. A	KW793	**OR831147**	**OR831191**	**OR941446**	**OR908948**	This paper
	South Korea—South—Jeju st. A	KW794	**OR831148**	**OR831192**	**OR941445**	**OR908950**	This paper
***Meioglossus maldivensis* sp. nov**	Maldives—South—Gaafu Dhaalu	KW878	**OR831159**	**OR831185**	–	**OR908938**	This paper
	Maldives—South—Gaafu Dhaalu	KW881	**OR831160**	**OR831186**	**OR941439**	–	This paper
	Maldives—South—Gaafu Dhaalu	KW882	**OR831161**	**OR831182**	**OR941440**	–	This paper
	Maldives—South—Gaafu Dhaalu	KW883	**OR831162**	**OR831187**	**OR941441**	–	This paper
***Meioglossus turkensis* sp. nov**	Turks & Caicos—West—Providenciales	KW803	**OR831157**	OR831175*	**OR941450**	**OR908940**	This paper
	Turks & Caicos—West—Providenciales	KW804	**OR831158**	**OR831176**	**OR941451**	**OR908941**	This paper
	Cuba—Northeast—Gibara	KW700	**OR831137**	–	**OR941448**	–	This paper
	Cuba—Northeast—Gibara	KW792	**OR831146**	**OR831177**	**OR941449**	**OR908939**	This paper

New sequences for this paper are shown in bold.

*Only first ~ 900 bp obtained.

**Only last ~ 900 bp obtained.

Sequences were aligned using MAFFT v7.450^55,56^ as implemented in Geneious. The E-INS-i algorithm was selected for ribosomal markers (16S and 18S rRNA), and the G-INS-i one for protein coding genes (COI and H3). Default parameters were selected for all alignments (Gap open penalty: 1.53, Offset value: 0.123, Scoring matrix: 200PAM/k = 2). Ribosomal gene datasets were re-aligned with the ‘nwildcard’ option selected on the MAFFT v7 online platform^57,58^, to ensure that missing data were not designated as gaps. As they show no variation in length, protein-coding gene alignments were trivial. However, to verify the presence of stop codons and indels, they were translated into amino-acid and re-aligned in Geneious. Single-gene datasets were concatenated using the ‘Concatenate Sequences or Alignments’ tool in Geneious. A total of 137 sequences were produced from the *Meioglossus* individuals. 37 sequences were generated for 16S rRNA, 33 sequences for 18S rRNA anterior fragment and 23 for the posterior fragment, 27 sequences for COI and 17 sequences for H3. The final combined dataset (16S rRNA, 18S rRNA, COI and H3) alignment is 3506 nucleotides in length (16S rRNA = 617 nucleotides, 18S rRNA = 1825 nucleotides, COI = 687 nucleotides and H3 = 377).

### Phylogenetic reconstructions

Phylogenetic reconstructions were performed on single and combined gene datasets using both Maximum Likelihood (ML) and Bayesian Inference (BI) methods.

ML analyses were conducted using RAxML v.8.2.11 (Randomized Axelerated Maximum Likelihood)^59^, as implemented in Geneious. RAxML only implements the General Time Reversible model (GTR). Given that and by performing the ModelTest program in Geneious using the Akaike Information Criterion (AIC), a GTR model with corrections for discrete gamma distribution (GTR + G + Ґ) was specified for the 16S rRNA, 18S rRNA and concatenated genes datasets, while a GTR + G model was selected for the protein coding genes. Nodal support estimations were generated using non-parametric bootstrapping with 1000 replicates.

Bayesian analyses were conducted using the MrBayes v3.2.6 plugin in Geneious^60^. Prior to analyses, optimal evolutionary models were inferred using JModelTest^61^ under the Schwartz Bayesian Information Criterion (BIC) on the CIPRES Sciences Gateway^62^ and were as follows: GTR + G for 16S rRNA; Hasegawa, Kishino and Yano (HKY + G) for 18S rRNA; HKY + G + Ґ for COI; GTR + G + Ґ for H3 and the concatenated dataset. Independent analyses were run twice on single gene and concatenated datasets, for 15 million generations with trees sampled every 1000 generations and using four heated chains. One quarter—corresponding to 3,750,000—of the generations were discarded as burn-in. Haplotype networks (only shown in Supplementary Figs. 12, 13) were generated on PopART v1.7^63^ using the Minimum Spanning Network^64^.

### Species delineation

Four methods widely used in species delineation were conducted; two based-tree methods; Generalized Mixed Yule Coalescent approach (GMYC)^65^ and Poisson Tree Process including a bayesian implementation (bPTP)^66^ and two genetic distances methods; Automatic Barcode Gap Discovery (ABGD)^67^ and Assemble Species by Automatic Partitioning (ASAP)^68^. Prior to analyses, outgroups were removed from all the datasets. No delineation analyses were conducted using either the 18S rRNA or H3 datasets, due to the small variability between the sequences, or the lack of data.

For GMYC analyses, ultrametric trees were generated using BEAST v2.6.7^69^. Parameters for the BEAST runs were defined with bEAUti v2.6.7, generating xml files. For all analyses, tree parameters were chosen based on a Yule model with a constant clock evolution. Nucleotide substitution models were estimated under AIC using JModelTest as implemented on CIPRES. 16S rRNA dataset was run under a Generalized Time Reversible model with a proportion of invariable sites (GTR + I), and HKY + G was selected for the COI dataset. Single and concatenated datasets underwent independent Markov Chain Monte Carlo (MCMC) analyses comprising 10 million generations with tree subsampling occurring every 1,000 generations. Convergence verification of all MCMC runs was done with Tracer v1.2.7^70^. A maximum clade credibility (MCC) consensus tree was obtained for each BEAST dataset with TreeAnnotator v2.6.7 to summarize Bayesian results. GMYC analyses were performed with RStudio 2022.02.3 (R Core Team, 2022) using the SPLITS v1.0–20 package^71^. bPTP analyses were carried out on the bPTP online server (https://species.h-its.org). Default parameters (MCMC generations of 100,000 and subsample of 100) were set, except for the burn-in set at 25%.

For ABGD delineation analyses, single gene dataset of COI and 16S were uploaded on the online platform (https://bioinfo.mnhn.fr/abi/public/abgd/). The Kimura model (K80) was selected for both analyses. Default parameters were used for the analyses (prior maximum divergence of intraspecific diversity P, Pmin = 0.001 and Pmax = 0.1/relative gap width X = 1.5). ASAP delineation method was performed using the ASAP online platform (https://bioinfo.mnhn.fr/abi/public/asap/asapweb.html). Again, the Kimura model was selected.

### Morphological observations

To test for potential morphological diagnostic traits, specimens were photographed and measured using an Olympus DP73 camera mounted on an Olympus IX70 inverted light microscope. Moreover, when material was available, we used immunohistochemical staining and Confocal Laser Scanning Microscopy (CLSM) to visualize ciliary patterns and neural architecture (tyrosinated tubulin- or acetylated α-tubulin immunoreactivity) and specific number and distribution of serotonin-like or FMRF-like immunoreactive somata (ser-LIR/ FMRF-LIR) (Table 3). These new morphological assessments complement the many previous morphological studies conducted on *Meioglossus* specimens from Belize and Bermuda^11^.

CLSM studies were done on material fixed in 4% paraformaldehyde in phosphate-buffered saline buffer (PBS) with 7% sucrose. Nine specimens of *Meioglossus* from six localities were examined (Cuba Chivirico and Miramar, Belize station 4, Eilat station 32, Maldives and South Korea station 19). Fixed specimens were rinsed twice in PBS and pre-incubated 2 h in PTA buffer (PBS with 1% Triton X-100, 0.25% bovine serum albumin and 7% sucrose). They were later incubated for 36 h in two primary antibodies, either monoclonal mouse anti-tyr-tubulin (SIGMA, T9028) or monoclonal mouse anti-acetylated α-tubulin (SIGMA, T6793) together with either polyclonal rabbit anti-serotonin (Sigma-Aldrich, S5545) or polyclonal rabbit anti-FMRF, with a final concentration of 1:400 in PTA. Specimens were thereafter rinsed in PBS six times over 2 h and incubated for 48 h in the secondary antibodies anti-rabbit-CY3 (Sigma-Aldrich, T5268) and anti-mouse-CY5 (Jackson ImmunoResearch, 115-175-146) with a final concentration of 1:400 in PTA. After the incubation, specimens were rinsed 3 times in PBS over 2 h and transferred through a graded series of increasing concentration of Vectashield^®^ mounting medium with DAPI (Vector Laboratories, VTECH-1200), before being whole-mounted on slides. Specimens were scanned using an Olympus FluoView FV-1000 CLSM. Sections, slices, and maximum intensity z-stack projection images were generated using the Imarisx64 v7.6.5 software (Oxford Instrument 2022).

Images and plates were arranged using Photoshop and Illustrator (Adobe Illustrator CS4 v14.0.0 and Adobe Photoshop CS4 v11.0). General morphology of *Meioglossus* is presented in Fig. 4. Supplementary Figs. 5–11 present detailed plates for each of the species, except for *Meioglossus* from Eilat st. 16 where the limited material was not adequately preserved for morphology.

## Results

### Phylogenetic reconstructions

**Figure 2:**
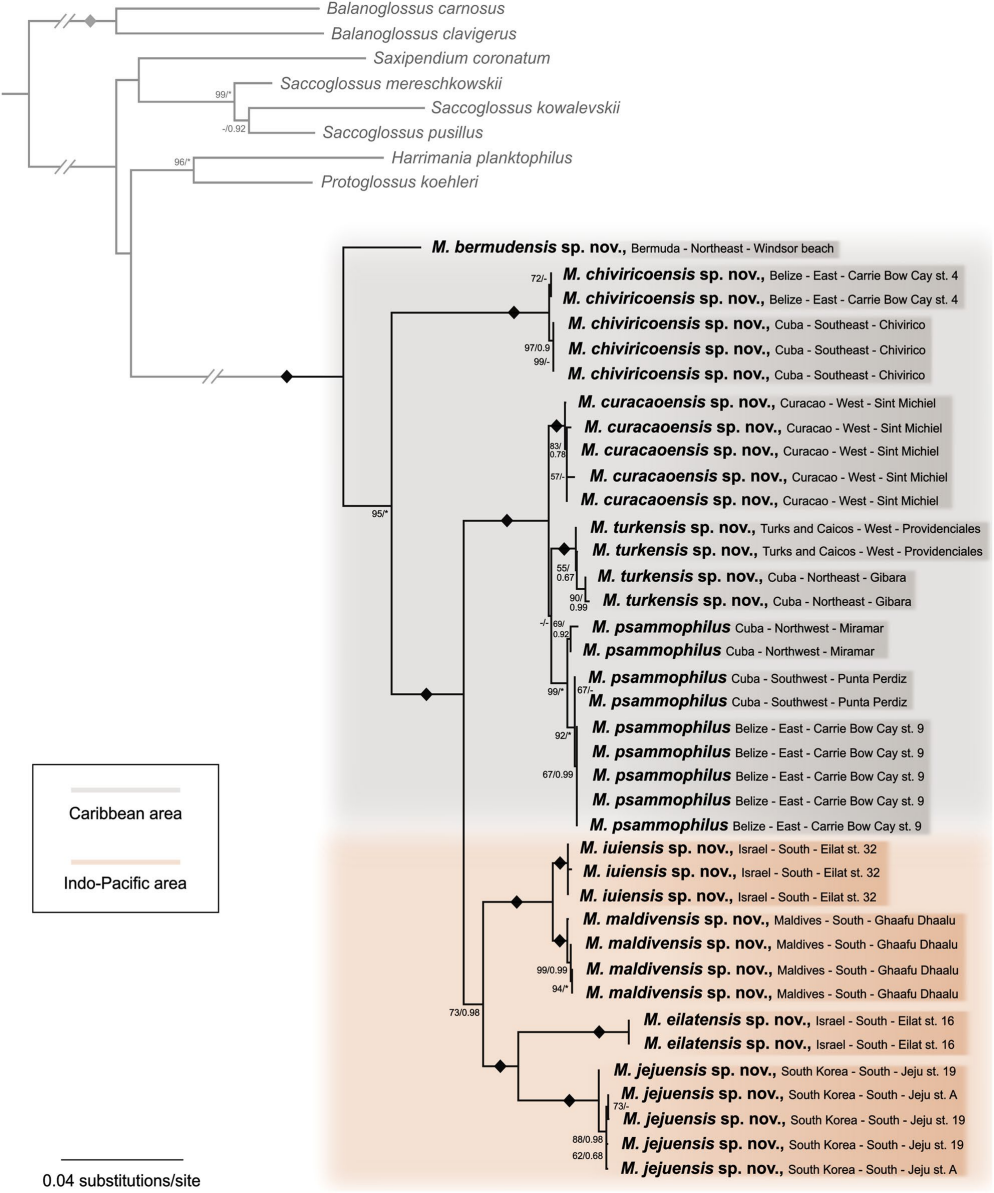
Phylogenetic relationships of *Meioglossus* using the concatenated gene datasets (16S rRNA, 18S rRNA, COI, H3). Topology based on Maximum Likelihood (ML) analyses of concatenated gene datasets. Nodal support is indicated with both Maximum Likelihood Bootstrapping (BS) and Bayesian Posterior Probabilities of the consensus tree (PP). Only nodal supports above BS > 50% or PP > 0.5 are shown. Those falling below this threshold are represented by a dash (–). Asterisks indicate maximum support in either BS = 100% or PP = 1. Diamond (◆) shapes indicates full support in both analyses.

Phylogenetic reconstructions confirmed the meiofaunal *Meioglossus* as a fully supported monophyletic clade (Bayesian Inference Posterior Probability PP = 1, Maximum Likelihood bootstrap BS = 100%) (Fig. 2).

The Bayesian inference (BI) and Maximum Likelihood (ML) reconstructions of the combined gene dataset present a similar topology with four major clades; three containing Caribbean individuals and one clade exclusively containing individuals from the Indo-Pacific area. Subclades within these are consistently found with high support, and each of the 14 sampled populations represent monophyletic clades except for mixing among the geographically close locations in South Korea. The only difference between the BI and ML reconstructions is the exact position of two specimens within their respective population clade, due to the low genetic variation within these populations.

The first of the four major clades is consistently found as sister branch to the remaining *Meioglossus,* cautioning that sequences were only obtained from one individual from Bermuda. A second larger fully supported clade branches off as sister to the remaining two clades and comprise individuals from Cuba (Chivirico) and Belize (station 4). Of the last two sister clades, one clade consists of three distinct subclades: (1) specimens from Curaçao gathering as a fully supported subclade, (2) specimens from Northwest and Southwest Cuba (Miramar and Punta Perdiz) and Belize (station 9) (subclade PP = 1.00 and BS = 99%), and (3) a fully supported subclade regrouping specimens from Northeast Cuba (Gibara) and Turks and Caicos Islands. The remaining major and exclusively Indo-Pacific clade, positioned among the Caribbean clades, brings together *Meioglossus* from Israel, South Korea, and the Maldives. Although moderately supported (PP = 0.98 and BS = 73%) this clade is consistently found in all concatenated genes tree analyses. The fully supported subclade from the Maldives is sister group to a fully supported subclade of specimens of Eilat station 32. A subclade of *Meioglossus* from the other Eilat locality (station 16) surprisingly nests next to a subclade of South Korean specimens from two localities (all clades fully supported) (Fig. 2).

Among all single-gene datasets analyses, the 16S rRNA dataset generates the most resolved and robust tree with similar topology to that of the combined-gene dataset and among the BI and ML reconstructions. The analyses of the COI dataset were also informative at the distant nodes although with less resolution and support of the major clades. Both 18S rRNA and H3 datasets were less complete and showed less variation among and within populations hereof yielding less resolved trees though with well supported major clades (single gene trees are shown in Supplementary Figs. 1–4).

### Species delineation

**Figure 3:**
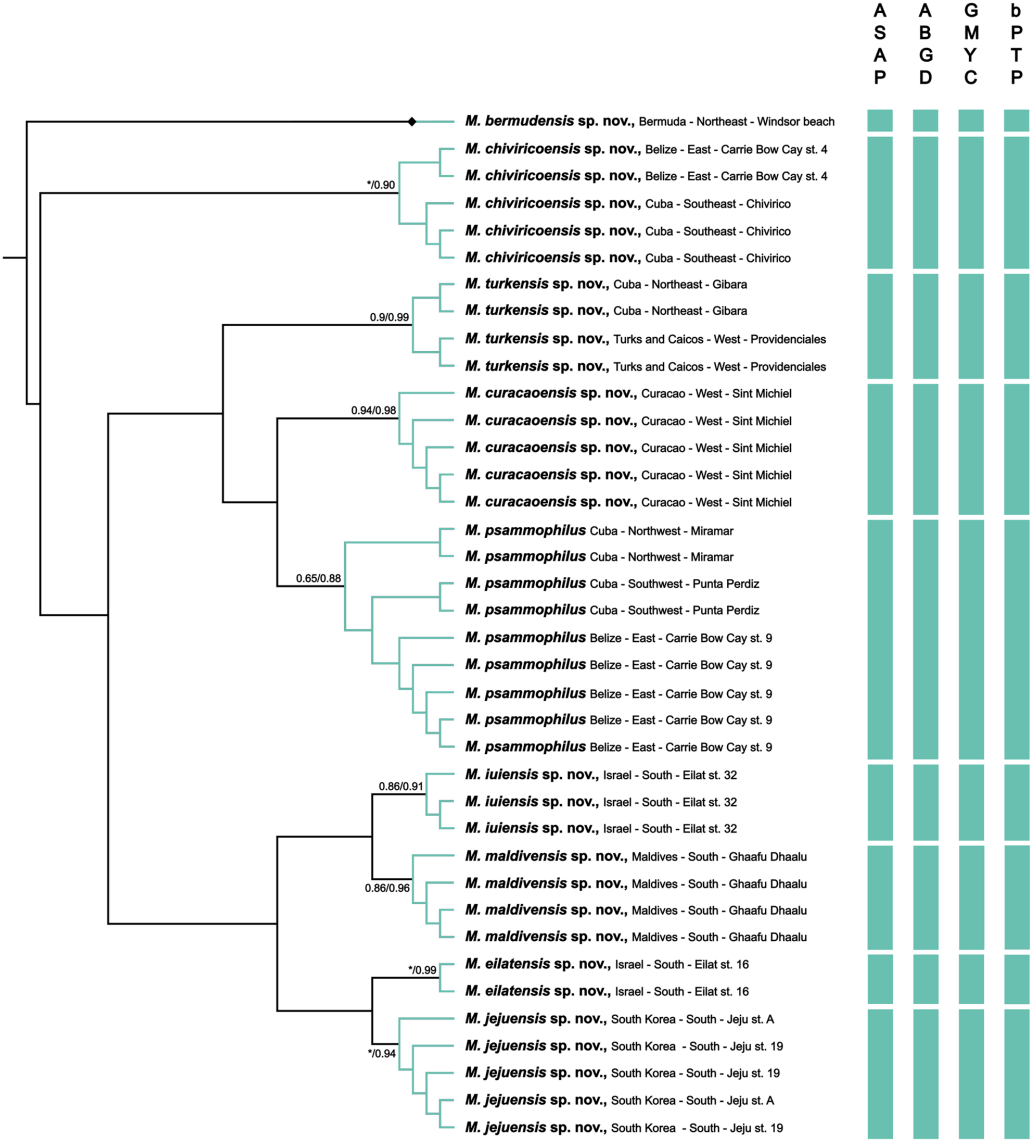
Ultrametric tree generated with BEAST, using the combined four-genes datasets (16S rRNA, 18S rRNA, COI, H3). Results of species delineations are indicated by vertical blue bars under each method used. Species hypotheses supports for both GMYC and bPTP are indicated (GMYC/bPTP). Asterisks indicate maximum support in one of the analyses and Diamond (◆) shapes indicate full support in both analyses.

All delineation analyses throughout the various datasets consistently yielded at least nine distinct phylogenetic entities (Table 2, Fig. 3), reflecting possible unique species.

**Table 2:** Species delineation results of *Meioglossus* using ABGD and ASAP methods for single datasets of 16S and COI (18S and H3 are omitted due to incomplete coverage and limited variation), bPTP and GMYC methods for both single and concatenated datasets.

Gene dataset	ABGD	ASAP	bPTP		GMYC		
	Est. ent	Est. ent	Est. ent (C.I.)	Mean	ML entities (C.I.)	Likelihood ratio	*P*
16S	9	9	9 (9–15)	10.15	9 (9–10)	32.42027	9.12e − 08***
COI	9	9	9 (8–15)	9.98	9 (9–10)	23.36372	8.45e − 06***
16S + COI	na	na	9 (9–14)	9.58	10 (9–11)	31.40941	1.51e − 07***
16S + 18S + COI + H3	na	na	9 (8–14)	9.50	9 (8–10)	17.14535	0.0002***

Abbreviations: C.I., confidence interval; ent, entities; Est, estimated; ML, maximum likelihood; na, not applicable.

***P ≤ 0.001.

Species delineation analyses ABGD and ASAP were only calculated for the single gene datasets of 16S rRNA and COI since the nuclear gene 18S rRNA presented poor variation between sequences, and the H3 dataset had a high level of missing data compared to the other datasets. Partitions with the lowest ASAP-score were chosen (2.5 for 16S and 2 for COI).

Species delineation analyses with bPTP and GMYC were performed using the single gene 16S rRNA and COI datasets as well as the combined four-genes dataset. All bPTP and GMYC analyses were statistically significant (*P* ≤ 0.05) (Table 2) and always recovered a minimum of nine entities. These entities were highly supported in the BEAST trees (Fig. 3) and congruent with the similarly highly supported clades in the phylogenetic analyses (Fig. 2).

### Morphological observations

To the extent preserved material allowed for, the external morphology and internal anatomy of *Meioglossus* was investigated using light microscopy and a combination of immunostaining and CLSM (Fig. 4, Supplementary Figs. 5–11).

**Figure 4:**
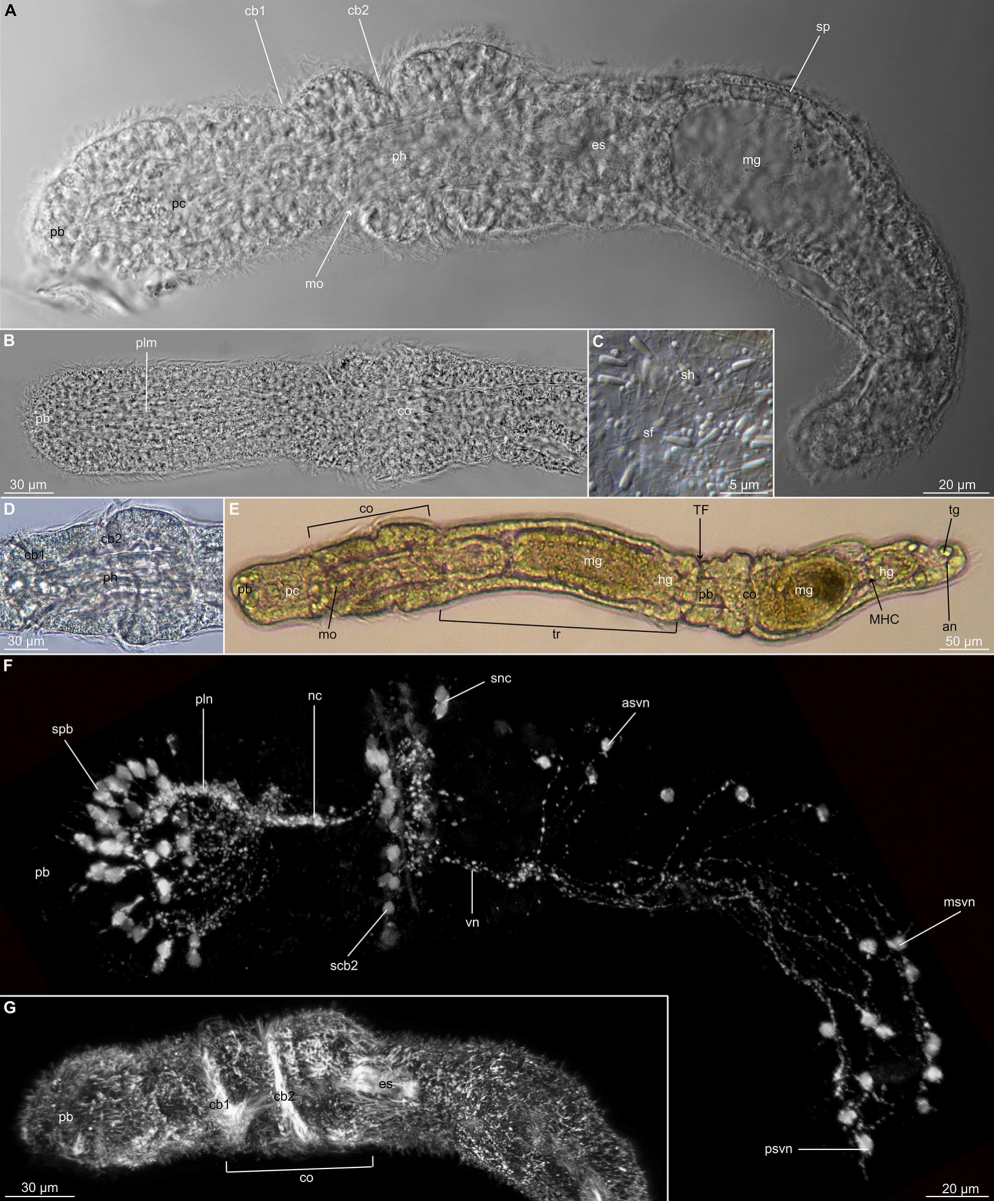
Overall morphological traits of *Meioglossus*, light- and confocal micrographs (LM and CLSM). (**A**). Overview of *M. chiviricoensis* sp. nov., lateral view, LM (NHMD-1731259). (**B**). Close-up of a *M. jejuensis* sp. nov. proboscis, lateral view, LM (NHMD-1731269). (**C**). Sperm heads and flagella from *M. psammophilus*, LM. (**D**). Close-up of *M. bermudensis* sp. nov. collar, dorsal view, LM. (**E**). *M. bermudensis* sp. nov. showing paratomy, dorsal view, LM. (**F**). Whole specimen of *M. psammophilus*, serotonin-LIR (ser-LIR), lateral view, CLSM (NHMD-90360). (**G**). Whole specimen of *M. chiviricoensis* sp. nov., Acetylated α-tub-LIR, lateral view, CLSM (NHMD-1731259). Abbreviations: an, anus; asvn; anterior somata of ventral nerve net; cb1, cb2, 1. & 2. Ciliary bands; co, collar region; es, esophagus; hg, hindgut; mg, midgut; MHC, midgut-hindgut constriction; mo, mouth opening; msvn, median somata of ventral nerve net; nc, neurochord (collar); pb, proboscis; pc, protocoel; ph, pharynx; plm, proboscis longitudinal muscles; pln, nerves innervating proboscis longitudinal muscles; psvn, posterior somata of ventral nerve net; sf, sperm flagellum; sh, sperm head; sp, sperm; scb2, somata of second ciliary band nerves; spb, somata of proboscis; snc, posterior somata of neurochord; TF, transverse fission zone; tr, trunk; tg, tail glands; vn, ventral nerve net (forms two ventral ‘cords’).

No new diagnostic traits could be defined based on LM observations, as specimens of all populations presented identical body-organization and detailed internal and external morphology. Even when comparing the specific location and numbers of serotonin-LIR somata (Table 3) among the five *Meioglossus* species examined with immunostaining and CLSM, no significant differences could be detected in the neural architecture. Measurements of body length (and relative length of body regions) did vary among the holotypes of the delineated species (Table 3). However, measurements of paratypes documented overlapping ranges between species and high intraspecific variations, due to different degree of anaesthetization and fixation protocols as well as different states of asexual reproduction. Morphometric observations could therefore not be used to establish new diagnostic traits.

**Table 3:** Measurements of *Meioglossus* species and comparison of the number of ser-LIR somata observed in five species of *Meioglossus*.

Species	Total length	Proboscis		Collar		Trunk		spb	scb2	snc	asvn + msvn + psvn
		Length	Width	Length	Width	Length	Width				
*M. psammophilus*	611 (455–611)	170 (103–178)	89 (62–91)	115 (67–150)	115 (75–115)	326 (130–326)	115 (73–115)	Est. 30	Est. 20	6	20–24
*M. bermudensis* sp. nov	298 (274–375)	92	76	62	87	160	66	Est. 30	18	6	23
*M. chiviricoensis* sp. nov	323 (323–532)	68 (68–132)	53 (46–62)	72 (72–107)	54 (51–77)	183 (178–330)	55 (55–72)	–	–	–	–
*M. curacaoensis* sp. nov	306 (306–370)	95 (95–171)	77 (45–77)	94 (64–91)	94 (71–94)	120 (120–138)	67 (54–67)	–	–	–	–
*M. eilatensis* sp.nov	258	93	85	55	86	110	72	–	–	–	–
*M. iuiensis* sp. nov	548 (548–594)	137 (78–137)	71 (36–71)	79 (79–160)	59 (41–59)	358 (288–358)	77 (50–77)	–	–	–	–
*M. jejuensis* sp. nov	555 (442–555)	167 (158–167)	67 (57–67)	100 (93–100)	56 (56–77)	288 (191–288)	61 (55–63)	Est. 40–45	18	6	Est. 7 apvn
*M. maldivensis* sp. nov	379 (379–387)	132 (99–132)	87 (60–96)	91 (80–91)	88 (68–103)	156 (156–208)	66 (58–79)	Est. 45–50	16	6	22
*M. turkensis* sp. nov	640	126 (126–132)	74 (52–74)	76 (76–93)	113 (68–113)	165 (165–415)	71 (53–71)	–	–	–	–

Measurements of holotypes; measurement ranges of paratypes given in parentheses.

Abbreviations: asvn, anterior serotonin-like immunoreactive (ser-LIR) somata of ventral nerve net; Est., estimated; msvn, median ser-LIR somata of ventral nerve net; psvn, posterior ser-LIR somata of ventral nerve net; scb2, ser-LIR somata of second ciliary band nerves; snc, posterior ser-LIR somata of neurochord; spb, ser-LIR somata of proboscis.

Light microscopy observations confirmed the presence of sperm in many individuals, with similar position and overall structure as in *M. psammophilus*. It is remarkable that among all populations examined throughout this study females were never observed, similar to what was reported by Worsaae et al. (2012)^11^. Moreover, asexual reproduction by paratomy was likewise observed in all newly recorded populations.

### Species descriptions

Phylum Hemichordata Bateson, 1885

Class Enteropneusta Gegenbaur, 1870

Family Harrimaniidae Spengel, 1901

Genus ***Meioglossus*** Worsaae, Sterrer, Kaul-Strehlow, Hay-Schmidt & Giribet, 2012

Diagnosis (emended from Meioglossus psammophilus^11^). Body hyaline with yellow gut. Proboscis delineated posteriorly by circumferential ring of cilia. Protocoel extending most of length and width of proboscis; lined by one-fibre thick ring of longitudinal muscles, encircled by circular musculature. Collar with middle circumferential groove lined by ring of long cilia and epidermal glands. Dorsal collar cord extending subepidermally, neuropores absent. Anterior trunk with slight longitudinal depression mid-dorsally; one pair of anterior, dorso-lateral, unciliated gill pores. Posterior trunk with supraterminal anus and large terminal glands. Sperm in paired testes or free in metacoel. Females not observed; sexual reproduction unknown. Asexual reproduction by transverse fission, in the form of paratomy.

***Meioglossus psammophilus*** Worsaae, Sterrer, Kaul-Strehlow, Hay-Schmidt & Giribet, 2012

*Redescription* (Figure 4C,F, Supplementary Tables 1, 3, Fig. 1E,F. in Worsaae et al. (2012)^11^, Fig. 5A,C,D. in Worsaae et al. (2012)^11^, Fig. 6A,C,F. in Worsaae et al. (2012)^11^).

*Holotype.* (designated in Worsaae et al. (2012)^11^): Male, 611 µm long, as permanent wholemount (NHMD-90314). Eastern Belize, Carrie Bow Cay, station 9 (16.8021, − 88.0768), 14 m depth, coral sand at reef. Sampled on the 2010-01-20.

*Paratypes.* (some designated in Worsaae et al. (2012)^11^): 37 specimens (5 male specimens as permanent wholemounts (NHMD-90315 to NHMD-90319), 11 on SEM stubs (NHMD-90320 to NHMD-90330), 8 sectioned on slides or grids (NHMD-90340 to NHMD-90347), 9 embedded in resin (NHMD-90331 to NHMD-90339), 2 vials with EtOH-fixed specimens (NHMD-90349 and NHMD-90355), 2 vials with aldehyde-fixed specimens(NHMD-90350 and NHMD-90351)), same sampling data as holotype. One male specimen as permanent wholemount (NHMD-1731257) Northwest Cuba, Miramar, La Habana (19.9829, − 75.8665), 15–17 m, medium sand. Sampled on the 2014-01-11. No type designated from Southwest Cuba, Punta Perdiz (locality data can be found in Supplementary Table 1).

*Molecular diagnosis*. DNA extracted from nine individuals. Molecular diagnostic characters 16S rRNA: T(188), T(298), A(515).

*Distribution and habitats*. Fine to medium sand patches and in coralline sand patches among coral reef, infralittoral. Northwest and Southwest Cuba, and East Belize.

*Remarks*. The validity of this redescribed species is supported by phylogenetic and species delineation analyses as well as from showing less interspecific than intra-specific similarity in 16S and CO1. In addition to specimens originally described as *M. psammophilus* from Belize station 9, new specimens sampled in the Southwest (Punta Perdiz) and Northwest (Miramar) of Cuba are here determined as *M. psammophilus* (Fig. 3). Several of the specimens comprised in the original description are here given a new name. Specimens sampled in Bermuda and previously identified as *M. psammophilus* are here shown to be *Meioglossus bermudensis* sp. nov. Specimens sampled in East Belize station 4 and previously identified as *M. psammophilus* are now shown to be *Meioglossus chiviricoensis* sp. nov.

Similarity matrix (See Supplementary Tables 4, 5 for details).

Intrapopulation similarity: similarity matrix of 16S rRNA shows 100% similarity among specimens from Belize st. 9, 100% similarity among specimens from Punta Perdiz, 99.92% similarity among specimens from Miramar.

Intraspecific similarities: similarity matrix of 16S rRNA shows 98.90–100% similarity among *M. psammophilus* specimens, and 99.00% similarity for COI.

Interspecific similarities: similarity matrix of 16S rRNA shows 96.44–96.84% similarity between *M. psammophilus* specimens and specimens of its sister species, *M. curacaoensis* sp. nov., and 96.45–97.35% similarity for COI.

***Meioglosus bermudensis*** sp. nov. Worsaae, 2024 (Figure 4D,E, Supplementary Tables 1, 3, Supplementary Fig. 5, Fig. 5B,E–G. in Worsaae et al. (2012)^11^, Fig. 6B,D,E,G,H. in Worsaae et al. (2012)^11^).

urn:lsid:zoobank.org:act:EA2259C4-EA98-4E87-879A-D775B516CB4E

*Holotype*. Specimen, 298 µm long as permanent wholemount (NHMD-90356), no sperm observed. Northeast Bermuda, Surf Bay, Windsor Beach (32.3317, − 64.6767), 12 m depth, patches of fine sand in the middle of reef with sheltered part of finer sand. Sampled on the 2007-10-18.

*Paratypes*. 40 specimens (24 specimens as permanent wholemounts (NHMD-90357 to NHMD-90380), 5 on SEM stubs (NHMD-90381 to NHMD-90385), 5 vials with EtOH-fixed specimens (NHMD-90392 to NHMD-90396), 6 vials with paraformaldehyde-fixed specimens (NHMD-90386 to NHMD-90391)), no sperm observed. Same sampling data as holotype.

*Molecular diagnosis*. Similar to Worsaae et al. (2012)^11^. DNA extracted from one individual.

*Etymology.* Named after the type locality ‘Bermuda’, and the Latin root *-ensis* (‘of’).

*Distribution and habitats.* Sand patched in middle reef with sheltered part of finer sand, infralittoral, Northeast Bermuda.

*Remarks*. The presence of a single individual in the analyses led to debate about whether to assign a name to this species discovered in Bermuda. However, all four markers sequenced for this specimen, consistently showed it to be a unique entity, branching off as sister group to all other *Meioglossus* spp. Its validity is further supported by the species delineation analyses and from showing high interspecific differences.

Similarity matrix (See Supplementary Tables 4, 5 for details).

Interspecific similarities: similarity matrix of 16S rRNA shows 80.44–83.25% similarity between *M. bermudensis* sp. nov specimens and those of its sister group, and 88.89–88.99% similarity for COI. The sister group of *M. bermudensis* sp. nov. comprise all other *Meioglossus* species.

***Meioglossus chiviricoensis*** sp. nov. Worsaae, 2024 (Fig. 4A,G, Supplementary Tables 1, 3, Supplementary Fig. 6).

urn:lsid:zoobank.org:act:D8F321BD-9A1D-46D2-9643-73377DE43EF4

*Holotype*. Male, 323 µm long, as permanent wholemount (NHMD-1731259). Southeast Cuba, Chivirico, El Laberinto (19.9686, − 76.4084), 29 m depth, coralline sand between coral reefs. Sampled on the 2014-11-23.

*Paratypes*. Four specimens as permanent wholemount (2 male; NHMD-1731258 and NHMD-1731260, and two specimens with no sperm observed; NHMD-1731261 and NHMD-1731262). Same sampling data as holotype.

*Molecular diagnosis.* DNA extracted from five individuals. Molecular diagnostic characters 16S rRNA: A(21), A(51), A(52), C(55), T(59), T(63), C(111), C(160), T(174), A(218), A(246), T(252), A(258), A(290), A(291), C(296), C(303), T(328), T(330), C(350), C(364), C(387), T(393), A(398), G(404), C(410), T(426), G(433), A(435), C(446), G(449), T(452), A(461), A(466), A(550), C(575).

*Etymology*. Named after the type locality near the city ‘Chivirico’, and the Latin root *-ensis* (‘of’).

*Distribution and habitats*. Coralline sand patches among coral reef, infralittoral. Southeast Cuba and East Belize.

*Remarks*. The validity of this species is supported by both phylogenetic and species delineation analyses, and from showing less interspecific than intra-specific similarity in 16S and CO1.

Similarity matrix (See Supplementary Tables 4, 5 for details).

Intrapopulation similarity: similarity matrix of both COI and 16S rRNA shows 100% similarity among specimens from Chivirico.

Intraspecific similarities: similarity matrix of 16S rRNA shows 99.49–100% similarity among *M. chiviricoensis* sp. nov. specimens, and 99.85–100% similarity for COI.

Interspecific similarities: similarity matrix of 16S rRNA shows 80.03–82.75% similarity between *M. chiviricoensis* sp. nov. specimens and those of its sister group, and 84.13–87.91% similarity for COI. The sister group of *M. chiviricoensis* sp. nov. comprises all *Meioglossus* species except *M. bermudensis* sp. nov.

***Meioglossus curacaoensis*** sp. nov. Worsaae, 2024 (Supplementary Tables 1, 3, Supplementary Fig. 7).

urn:lsid:zoobank.org:act:9F2F4481-3DE0-4936-975E-F2FDC011E086

*Holotype*. 306 µm long EtOH-fixed specimen (NHMD-1731263), no sperm observed. West Curaçao, Sint Michiel (12.1479, − 68.9990), 0.3 m depth, coral sand. Sampled on the 2018-04-21.

*Paratypes*. Two EtOH-fixed specimen (NHMD-1731264 and NHMD-1731265), no sperm observed. Same sampling data as holotype.

*Molecular diagnosis.* DNA extracted from five individuals. No CLSM observations on neural characters obtained. Molecular diagnostic characters 16S rRNA: A(299), G(304).

*Etymology.* Named after the type locality ‘Curaçao’, and the Latin root *-ensis* (‘of’).

*Distribution and habitats*. Coral sand, infralittoral, West Curaçao.

*Remarks*. The validity of this species is supported by both phylogenetic and species delineation analyses, and from showing less interspecific than intra-specific similarity in 16S and CO1.

Similarity matrix (See Supplementary Tables 4, 5 for details).

Intraspecific similarities: similarity matrix of 16S rRNA shows 100% similarity among *M. curacaoensis* sp. nov. specimens, and 99.85–100% similarity for COI.

Interpecific similarities: similarity matrix of 16S rRNA shows 96.44–96.84% similarity between *M. curacaoensis* sp. nov. specimens and those of its sister group, *M. psammophilus,* and 96.45–97.35% similarity for COI.

***Meioglossus eilatensis*** sp. nov. Worsaae, 2024 (Supplementary Tables 1, 3).

urn:lsid:zoobank.org:act:537F17D0-9BA9-4209-9D4E-DFE659A410FF

*Holotype*. 258 µm long EtOH-fixed specimen (NHMD-1731266), no sperm observed. Southern Israel, Eilat, near the Inter-University for Marine Sciences of Eilat, station 16 (29.5164, 34.9284), at 15–18 m depth, medium to coarse sand with seagrass (*Halophila*). Sampled the 2014–02-11.

*Molecular diagnosis.* No morphological observations obtained. DNA extracted from two individuals. Molecular diagnostic characters 16S rRNA: G(152), T(246), T(436).

*Etymology*. Named after the type locality ‘Eilat’, and the Latin root *-ensis* (‘of’).

*Distribution and habitats*. Medium to coarse sand with seagrass (*Halophila*), infralittoral, Southern Israel.

*Remarks*: The validity of this species is supported by both phylogenetic and species delineation analyses, and from showing less interspecific than intra-specific similarity in 16S and CO1.

Similarity matrix (See Supplementary Tables 4, 5 for details).

Intraspecific similarities: similarity matrix of 16S rRNA shows 100% similarity among *M. eilatensis* sp. nov. CO1 only obtained from one individual.

Interspecific similarities: similarity matrix of 16S rRNA shows 87.58% similarity between *M. eilatensis* sp. nov. specimens and those of its sister group, *M. jejuensis* sp. nov, and 86.15–87.44% similarity for COI.

***Meioglossus iuiensis*** sp. nov. Worsaae, 2024 (Supplementary Tables 1, 3, Supplementary Fig. 8).

urn:lsid:zoobank.org:act:7899FF09-556F-4643-B4BF-F19A2C119921

*Holotype.* 548 µm long specimen as permanent wholemount (NHMD-1731267), no sperm observed. Southern Israel, Eilat, near the Inter-University for Marine Sciences of Eilat, station 32 (29.5059, 34.9196), 8 m depth, coral sand. Sampled on the 2014–02-18.

*Paratype.* One specimen as permanent wholemount (NHMD-1731268). Same sampling data as holotype.

*Molecular diagnosis.* DNA extracted from three individuals. Molecular diagnostic characters 16S rRNA: C(141), C(36), C(50), T(53), A(63), T(171), G(186), A(251), T(266), A(270), T(288), T(289), G(296), T(306), A(315), T(325), T(342), T(365), A(369), T(380), A(384), G(410).

*Etymology*. Named after the type locality ‘IUI’, the Inter-University Institute for Marine Sciences of Eilat, and from the Latin root *-ensis*, meaning ‘originated in’.

*Distribution and habitats*. Coral sand, infralittoral, Southern Israel.

*Remarks*. The validity of this species is supported by both phylogenetic and species delineation analyses, and from showing less interspecific than intra-specific similarity in 16S and CO1.

Similarity matrix (See Supplementary Tables 4, 5 for details).

Intraspecific similarities: similarity matrix of 16S rRNA shows 100% similarity among *M. iuiensis* sp. nov., and 99.41–99.55% similarity for COI.

Interspecific similarities: similarity matrix of 16S rRNA shows 96.89–96.98% similarity between *M. iuiensis* sp. nov. specimens and those of its sister group, *M. maldivensis* sp. nov., and 97.56–98.07% similarity for COI.

***Meioglossus jejuensis*** sp. nov. Worsaae, 2024. (Fig. 4B, Supplementary Tables 1, 3, Supplementary Fig. 9).

urn:lsid:zoobank.org:act:8E93E4E1-E735-48E6-8F3E-14860629A611

*Holotype*. 555 µm long specimen as permanent wholemount (NHMD-1731269), no sperm observed. Southern South Korea, Jeju Island, Seopseom Islet, station 19 (33.2294, 126.6027), 33 m depth, shell gravel. Sampled on the 2015–10-19.

*Paratypes*. One paraformaldehyde-fixed specimen (NIBRIV0000910960). Southern South Korea, Jeju Island, Seopseom Islet, station B (33.2304, 126.6015), 15 m depth, shell gravel. Sampled on the 2018–05-25. One glutaraldehyde-fixed specimen (NHMD-1731270), same sampling data as holotype. Three glutaraldehyde-fixed specimens (NHMD-1731271 to NHMD-1731273). Southern South Korea, Jeju Island, Seopseom Islet, station A (33.2304, 126.6015), 14 m depth, shell gravel. Sampled on the 2018-05-28. No sperm observed in these specimens.

*Molecular diagnosis.* DNA extracted from five individuals. Molecular diagnostic characters 16S rRNA: T(13), T(79), T(183), T(208), T(245), C(245), C(248), G(281), G(282), C(293), C(332), T(333), T(334), G(374), G(425), T(551), G(572), C(580).

*Etymology*. Named after the type locality ‘Jeju’, and the Latin root *-ensis* (‘of’).

*Distribution and habitats*. Shell gravel, infralittoral, Southern South Korea.

*Remarks*. The validity of this species is supported by both phylogenetic and species delineation analyses, and from showing less interspecific than intra-specific similarity in 16S and CO1.

Similarity matrix (See Supplementary Tables 4, 5 for details).

Intrapopulation similarity: similarity matrix of 16S rRNA shows 100% similarity among specimens from South Korea st. 19, and 100% among specimens from South Korea st. A. COI shows 98.56–100% similarity among specimens from South Korea st. 19, and 99.71% among specimens from South Korea st. A.

Intraspecific similarities: similarity matrix of 16S rRNA shows 100% similarity among *M. jejuensis* sp. nov., and 98.56–100% for COI.

Interspecific similarities: similarity matrix of 16S rRNA shows 87.58% similarity between *M. jejuensis* sp. nov. specimens and those of its sister group, *M. eilatensis* sp. nov., and 86.15–87.44% similarity for COI.

***Meioglossus maldivensis*** sp. nov. Worsaae, 2024 (Supplementary Tables 1, 3, Supplementary Fig. 10).

urn:lsid:zoobank.org:act:FFE4D44A-922B-4CC5-AA84-1C5D211AA6E7

*Holotype*. One EtOH-fixed specimen (NHMD-1731274), no sperm observed. Southern Maldives, Ghaafu Dhaalu Atoll, (0.1993, 73.2304), 10–14 m depth, coarse coral sand. Sampled on the 2021-11-22.

*Paratype*. One specimen as permanent wholemount (NHMD-1731275), no sperm observed. Southern Maldives, Ghaafu Dhaalu Atoll, (0.2275, 73.2131), 12.3 m depth, fine coral sand. Sampled on the 2021-11-30.

*Molecular diagnosis.* DNA extracted from four individuals. Molecular diagnostic characters 16S rRNA: G(1), T(134), G(205), G(207).

*Etymology*. Named after the type locality ‘Maldives’, and the Latin root *-ensis* (‘of’).

*Distribution and habitats*. Fine to coarse well sorted coral sand, infralittoral, Southern Maldives.

*Remarks*. The validity of this species is supported by both phylogenetic and species delineation analyses, and from showing less interspecific than intra-specific similarity in 16S and CO1.

Similarity matrix (See Supplementary Tables 4, 5 for details).

Intraspecific similarities: similarity matrix of 16S rRNA shows 99.69–100% similarity among *M. maldivensis* sp. nov., and 99.93–100% for COI.

Interspecific similarities: similarity matrix of 16S rRNA shows 96.89–96.98% similarity between *M. maldivensis* sp. nov. specimens and those of its sister group, *M. iuiensis* sp. nov., and 97.56–98.07% similarity for COI.

***Meioglossus turkensis*** sp. nov. Worsaae, 2024 (Supplementary Tables 1, 3, Supplementary Fig. 11).

urn:lsid:zoobank.org:act:FA842EAE-A2AB-4B38-B00A-917B2FC5C93C

*Holotype*. 367 µm long paraformaldehyde-fixed specimen (NHMD-1731276), no sperm observed. Western Turks and Caicos Islands, Providenciales, Smiths reefs (21.7898, -72,2273), 1.5 m depth, coarse heterogeneous coral rubble and sand. Sampled on the 2019–01-06.

*Paratypes*. One paraformaldehyde-fixed specimen (NHMD-1731277), no sperm observed. Same sampling data as holotype. No type designated from Northeast Cuba, Gibara (locality data can be found in Supplementary Table 1).

*Molecular diagnosis.* No CLSM observations on neural characters obtained. DNA extracted from four individuals. Molecular diagnostic characters 16S rRNA: T(15), A(264), C(291), T(394), G(434), A(566).

*Etymology*. Named after the type locality in the ‘Turks and Caicos Islands’, and the Latin root *-ensis* (‘of’).

*Distribution and habitats*. Medium to coarse heterogeneous coral rubble and sand, infralittoral. Western Turks and Caicos Islands, and Northeast Cuba.

*Remarks*. The validity of this species is supported by both phylogenetic and species delineation analyses, and from showing less interspecific than intra-specific similarity in 16S and CO1.

Similarity matrix (See Supplementary Tables 4, 5 for details).

Intrapopulation similarity: similarity matrix of 16S rRNA shows 100% similarity among specimens from Turks and Caicos Islands, and 99.83% among specimens from Gibara. COI shows 100% similarity among specimens from Turks and Caicos Islands, and 99.59% among specimens from Gibara.

Intraspecific similarities: similarity matrix of 16S rRNA shows 99.67–100% similarity among *M. turkensis* sp. nov. specimens, and 99.44–100% similarity for COI.

Interspecific similarities: similarity matrix of 16S rRNA shows 95.59–96.67% similarity between *M. turkensis* sp. nov. specimens and those of its sister group, and 96.37–97.35% similarity for COI. The sister group of *M. maldivensis* sp. nov. comprises *M. curacaoensis* sp. nov. and *M. psammophilus* specimens.

## Discussion

Our study highlights a hidden diversity within *Meioglossus* as found in several other meiofaunal genera from disparate taxa such as Annelida^18,26,72^, Nematoda^73^, Nemertea^28^, Rotifera^74–76^, Tardigrada^77,78^ and Mollusca^31,79^. Using multiple species delineation methods and a conservative estimate, we recovered nine separate entities of *Meioglossus* with relatively restricted geographic distribution.

Prior to this study only one meiofaunal species of enteropneust was described, the Caribbean *Meioglossus psammophilus*^11^. We not only document that species of *Meioglossus* can be found worldwide, but that these larval-looking microscopic enteropneusts constitute a monophyletic genus. Whether it is with nuclear or mitochondrial sequences, single or concatenated datasets, this study corroborates the monophyly of this genus.

None of the newly discovered *Meioglossus* species have revealed female individuals. Instead, they look morphologically identical to *M. psammophilus* and likewise contain sperm and reproduce asexually through paratomy^11^. The lack of specimens carrying eggs or embryos in any of the newly discovered populations questions the function of the sperm described in Worsaae et al. (2012)^11^. The seeming absence of females and sexual reproduction put relevance to the previously mentioned hypothesis in Worsaae et al. (2012)^11^, suggesting that the sperm-like structure could instead function as an energy reserve. These structures repeatedly found in most populations should be further investigated with advanced microscopy and staining methods to help address their detailed structure and correct interpretation as sperm. The repetitive finding of paratomy in most of these populations may also indicate that these species mainly (or only) reproduce asexually. If *Meioglossus* reproduces only by paratomy, this would to our knowledge be a unique example in the animal kingdom, where entirely asexual reproduction in a free-living solitary species otherwise normally involve parthenogenesis (including eggs)^80,81^. All these new findings have unfortunately not solved the enigma of the *Meioglossus* life cycle. However, our analyses showed a high cryptic species diversity and broad distribution of the genus. Furthermore, the absence of females in any of the newly discovered localities, indirectly support that species of *Meioglossus* are not just larvae or dwarf males of a macroscopic female but truly represent a broadly distributed genus of permanent meiofauna species.

In the more densely sampled Caribbean area we find several populations constituting one species. Examples are *M. psammophilus* gathering relatively distant populations from Belize (station 9), Northwest and Southwest Cuba, and *M. chiviricoensis* sp. nov. found in both Belize (station 4) and Southeast Cuba. *Meioglossus turkensis* sp. nov., comprises specimens from closer localities in Turks and Caicos and Northeast Cuba, respectively. *Meioglossus jejuensis* sp. nov. groups animals from even closer localities. Single species comprising several populations, and in particular the latter two distribution patterns, can be easily explained by transport of sediment and animals by shared currents across shallow waters not hindered by the lack of a larval or dormant dispersal stages^20,41,82–84^. Such passive dispersal mechanisms are also known to act on microscopic organisms over larger distances, potentially aided by rafting or drifting and by human activities such as ballast and transport sediment, or aquaculture^16,85–88^.

On the other hand, several of the *Meioglossus* populations represent individual species, even when sampled geographically close such as the two species in the Red Sea near Eilat. These overlapping distribution patterns of species upholding genetic disparity but having seemingly similar morphology, may reflect specific physiological adaptations to the abiotic properties of their respective environment (e.g., granulometry, current, temperature, salinity, oxygen, pH)^89,90^. For instance, the environmental conditions of the two Red Sea populations are strikingly different with one locality exhibiting very fine sandy sediment (housing *M. eilatensis*) and the other coarse coralline sand (housing *M. iuiensis*), possibly explaining the presence of two different species within close geographical range. The apparent lack of genetic mixing between some of these geographically close populations, e.g., in Eilat and Belize (haplotype networks are shown in Supplementary Figs. 12 and 13), could also reflect distinct reproductive properties, or even be the result of *Meioglossus* only reproducing asexually^91^.The Everything is Everywhere (EIE) hypothesis^92^ asserts an ubiquitous distribution of microscopic organisms^16^. None of the newly described *Meioglossus* species are globally distributed. These on the contrary, tend to be specific to particular geographical regions and hereby go against the EIE hypothesis. However, the broad and somewhat overlapping distribution of some of the Caribbean species (e.g., including specimens from Belize and Cuba) does indicate that *Meioglossus* can be dispersed relatively far—hereby partly supporting the EIE statement that ‘the environment selects’ whether they will successfully colonize these new areas^16,93,94^.

Morphological stasis is noteworthy in *Meioglossus*, showing genetic diversity without any distinct morphological differentiation. This phenomenon of morphological stability has been noticed in other meiobenthic groups, e.g., copepods, segmented worms, or sea slugs^79,95,96^. It has been suggested to reflect the harsh selection by the physical constraints of the interstitial habitat, such as the restricted space available between sand grains or the habitat instability due to currents or waves actions^21,79,97^. Morphological stasis might also be reinforced by irreversible gene loss^98^. Microscopic animal may not always possess small, nor compacted genomes, but miniaturization events could influence the genome composition. In this case, genes or gene families might be lost, pathways modified, and transposable elements vastly reduced. In the miniaturized, compacted genome of the meiofaunal annelid *Dimorphilus gyrociliatus* (Schmidt, 1857) ^16^, the loss of a few developmental genes could be related to morphological losses of e.g. chaetae (post1 and FGFligand) and reduced mesodermal derivatives such as coeloms (VEGF ligands)^22^. Moreover, despite our limited understanding of the effect of transposable element loss, it may have the potential to influence a species’ capacity for morphological diversification.

Findings of a total of 14 populations of *Meioglossus* from the West Atlantic, the Red Sea, the Indian Ocean, and the East China Sea, documents the broad distribution of *Meioglossus* and points to a circumtropical distribution of the genus. It is intriguing that *Meioglossus* exhibits such a wide distribution despite its simplified morphology and apparent absence of larval stages. The broad distribution might be attributed to *Meioglossus* being an old genus which potentially dispersed with tectonic plate movements and/or movement of shallow sediments with storms. As being the case for some other meiofaunal genera and families (e.g., Refs.^99,100^), *Meioglossus* seems limited to tropical and subtropical regions, since it has never been found in temperate or polar regions. Our phylogenetic investigation demarks an Indo-Pacific clade nested among the Caribbean clades, indicating that *Meioglossus* may have originated in the Caribbean/West Atlantic Ocean and later dispersed into the Indo-Pacific Oceans. However, it is cautioned that the Indo-Pacific clade is poorly supported, and that many tropical and subtropical areas, especially in the East Pacific, have never been explored for *Meioglossus*. Thus, potential new findings of *Meioglossus* may change our current understanding of the intrageneric relationship.

### Supplementary Information

Supplementary Information.

## Data Availability

The datasets generated and analyzed during the current study are available in GenBank database with the accession numbers OR831127-OR831162 for 16S rRNA, OR831163-OR831195 for 18S rRNA, OR941431-OR941457 for COI and OR908937-OR908953 for H3. Final alignments and raw trees can be found online in Figshare (10.6084/m9.figshare.25353082). This published work and the nomenclature acts it contains have been registered in ZooBank, the online registration system for the International Code of Zoological Nomenclature (ICZN). The ZooBank LSIDs (Life Science Identifiers) can be resolved and the associated information viewed through any standard web browser by appending the LSID to the prefix 'https://zoobank.org/'. the LSID for this publication is: urn:lsid:zoobank.org:pub:4FE32B90-D99B-4BB4-8EED-86A4A5FEC8F4.
