# Supplementary material for: Circumtropical distribution and cryptic species of the meiofaunal enteropneust Meioglossus (Harrimaniidae, Hemichordata)
Source: Sci Rep. 2024 Apr 23;14:9296. doi: 10.1038/s41598-024-57591-0 (PMC11039715; doi:10.1038/s41598-024-57591-0)
Supplement: Supplementary file 1 — Supplementary Information. [file 41598_2024_57591_MOESM1_ESM.pdf]

Circumtropical distribution and cryptic species of the meiofaunal enteropneust *Meioglossus* (Harrimaniidae, Hemichordata)

Éloïse Defourneaux[1], Maria Herranz[1,2], Maickel Armenteros[3], Martin V. Sørensen[4], Jon L. Norenburg[5], Taeseo Park[6], Katrine Worsaae[1*]

[1]Marine Biological Section, Department of Biology, University of Copenhagen, Universitetsparken 4, 2100-DK Copenhagen, Denmark
Éloïse Defourneaux, OrcID 0009-0006-3510-117X
Katrine Worsaae, OrcID 0000-0003-0443-4298

[2]Area of Biodiversity and Conservation, Superior School of Experimental Science and Technology (ESCET), Rey Juan Carlos University, C/ Tulipán S/N, 28933 Mostoles, Madrid, Spain. OrcID 0000-0002-3020-6072

[3]Unidad Académica Mazatlán, Instituto de Ciencias del Mar y Limnología, Universidad Nacional Autónoma de México. Av. Joel Montes Camarena S/N, 82040, Mazatlán, México. OrcID 0000-0002-9736-9247

[4]Natural History Museum Denmark, University of Copenhagen, Universitetsparken 15, 2100-DK Copenhagen, Denmark. OrcID 0000-0002-0377-0276

[5]Smithsonian National Museum of Natural History, Washington, DC, USA. OrcID 0000-0001-7776-1527

[6]Species Diversity Research Division, National Institute of Biological Resources, Hwangyeong-ro 42, Incheon 22689, South Korea. OrcID 0000-0003-2057-9411

Corresponding author*: kworsaae@bio.ku.dk

DOI: 10.1038/s41598-024-57591-0

Supplementary material

Supplementary Table 1. Locality and collection data of *Meioglossus* specimens used in our molecular study.

Species	ID extraction	*Meioglossus* specimens	Latitude (°)	Longitude (°)	Depth (m)	Sediment	Date (yyyy-mm-dd)
Meioglossus psammophilus	KW512	Cuba - Northwest - Miramar	19.9829	-75.8665	15-17	Medium coral sand	2014-01-11
	KW802	Cuba - Northwest - Miramar	19.9829	-75.8665	15-17	Medium coral sand	2014-01-11
	KW511	Cuba - Southwest - Punta Perdiz	22.1149	-81.1168	15-20	Fine-medium coral sand	2014-01-08
	KW546	Cuba - Southwest - Punta Perdiz	22.1149	-81.1168	15-20	Fine-medium coral sand	2014-01-08
	KW099 A	Belize - East - Carrie Bow Cay st. 9	16.8021	-88.0768	14	Coral sand at reef	2010-01-20
	KW099 B	Belize - East - Carrie Bow Cay st. 9	16.8021	-88.0768	14	Coral sand at reef	2010-01-20
	KW099 C	Belize - East - Carrie Bow Cay st. 9	16.8021	-88.0768	14	Coral sand at reef	2010-01-20
	KW099 D	Belize - East - Carrie Bow Cay st. 9	16.8021	-88.0768	14	Coral sand at reef	2010-01-20
	KW099 E	Belize - East - Carrie Bow Cay st. 9	16.8021	-88.0768	14	Coral sand at reef	2010-01-20
***Meioglossus bermudensis* sp. nov.**	KWB	Bermuda - Northeast - Windsor beach	32.3317	-64.6767	12	Fine coral sand patches in middle of reef	2007-10-18
***Meioglossus chiviricoensis* sp. nov.**	KW096 A	Belize - East - Carrie Bow Cay st. 4	16.8037	-88.0769	15	Coral sand at reef	2010-01-16
	KW096 B	Belize - East - Carrie Bow Cay st. 4	16.8037	-88.0769	15	Coral sand at reef	2010-01-16
	KW702	Cuba - Southeast - Chivirico	19.9686	-76.4084	29	Coralline sand in spur-and-groove biotope	2014-11-23
	KW785	Cuba - Southeast - Chivirico	19.9686	-76.4084	29	Coralline sand in spur-and-groove biotope	2014-11-23
	KW786	Cuba - Southeast - Chivirico	19.9686	-76.4084	29	Coralline sand in spur-and-groove biotope	2014-11-23
***Meioglossus curacaoensis* sp. nov**	KW795	Curaçao - West - Sint Michiel	12.1479	-68.9990	0.3	Coral sand	2018-04-21
	KW796	Curaçao - West - Sint Michiel	12.1479	-68.9990	0.3	Coral sand	2018-04-21
	KW797	Curaçao - West - Sint Michiel	12.1479	-68.9990	0.3	Coral sand	2018-04-21
	KW798	Curaçao - West - Sint Michiel	12.1479	-68.9990	0.3	Coral sand	2018-04-21
	KW799	Curaçao - West - Sint Michiel	12.1479	-68.9990	0.3	Coral sand	2018-04-21
***Meioglossus eilatensis* sp. nov.**	KW800	Israel - South - Eilat st. 16	29.5164	34.9284	15-18	Medium sand between sea grass *Halophila*	2014-02-11
	KW801	Israel - South - Eilat st. 16	29.5164	34.9284	15-18	Medium sand between sea grass *Halophila*	2014-02-11
***Meioglossus iuiensis* sp. nov.**	KW513	Israel - South - Eilat st. 32	29.5059	34.9169	8	Coral sand	2014-02-18
	KW790	Israel - South - Eilat st. 32	29.5059	34.9169	8	Coarse coral sand	2014-02-18
	KW791	Israel - South - Eilat st. 32	29.5059	34.9169	8	Coarse coral sand	2014-02-18
***Meioglossus jejuensis* sp. nov.**	KW718	South Korea - South - Jeju st. 19	33.2294	126.6027	33	Shell gravel	2015-10-19
	KW788	South Korea - South - Jeju st. 19	33.2294	126.6027	33	Shell gravel	2015-10-19
	KW789	South Korea - South - Jeju st. 19	33.2294	126.6027	33	Shell gravel	2015-10-19
	KW793	South Korea - South - Jeju st. A	33.2304	126.6015	14	Shell gravel	2018-05-28
	KW794	South Korea - South - Jeju st. A	33.2304	126.6015	14	Shell gravel	2018-05-28
***Meioglossus maldivensis* sp. nov.**	KW878	Maldives - South - Gaafu Dhaalu	0.1993	73.2304	10-14	Well sorted coarse coral sand	2021-11-22
	KW881	Maldives - South - Gaafu Dhaalu	0.1993	73.2304	10-14	Well sorted coarse coral sand	2021-11-22
	KW882	Maldives - South - Gaafu Dhaalu	0.1993	73.2304	10-14	Well sorted coarse coral sand	2021-11-22
	KW883	Maldives - South - Gaafu Dhaalu	0.1993	73.2304	10-14	Well sorted coarse coral sand	2021-11-22
***Meioglossus turkensis* sp. nov.**	KW803	Turks & Caicos - West - Providenciales	21.7898	-72.2273	1.5	Coarse heterogenous coral rubble and sand	2019-01-06
	KW804	Turks & Caicos - West - Providenciales	21.7898	-72.2273	1.5	Coarse heterogenous coral rubble and sand	2019-01-06
	KW700	Cuba - Northeast - Gibara	21.2105	-76.2424	18	Medium to coarse sand	2014-11-18
	KW792	Cuba - Northeast - Gibara	21.2105	-76.2424	18	Medium to coarse sand	2014-11-18

Supplementary Table 2. List of primers sequences and annealing temperatures used in PCR.

Primers	Direction	Annealing temperature	Primer sequence (5’ → 3’)	References
**16S**				
ar-L	Forward	45°C	CGC CTG TTT ATC AAA AAC AT	Palumbi, 1996[1]
br-H	Reverse		CCG GTC TGA ACT CAG ATC ACG T	Palumbi, 1996[1]
**18S 1st fragment**				
1F	Forward	49°C	TAC CTG GTT GAT CCT GCC AGT AG	Rousset et al., 2007[2]
5R	Reverse		CTT GGC AAA TGC TTT CGC	Rousset et al., 2007[2]
G51	Forward	49°C	GGT TGA TCC TGC CAG TAG	Hillis & Dixon, 1991[3]
G747	Reverse		CGG TAT CTG ATC GTC TTC GA	Hillis & Dixon, 1991[3]
**18S 3rd fragment**				
18Sa2.0	Forward	55°C	ATG GTT GCA AAG CTG AAA C	Whiting et al., 1997[4]
9R	Reverse		GAT CCT TCC GCA GGT TCA CCT AC	Giribet et al., 1996[5]
G952	Forward	49°C	GCC AAA GCA TTT GCC AAG MA	Cohen et al., 2004[6]
G944	Reverse		TGA TCC TTC TGC AGG TTC ACC TAC	Lovejoy & Potvin, 2011[7]
**COI**				
19F	Forward	45°C	CWA ATC AYA AAG ATA TTG GAA C	Colgan et al., 2001[8]
726R	Reverse		AAT ATA WAC TTC WGG GTG ACC	Colgan et al., 2001[8]
**H3**				
aF	Forward	53°C	ATG GCT CGT ACC AAG CAG ACV GC	Colgan et al., 1998[9]
aR	Reverse		ATA TCC TTR GGC ATR ATR GTG AC	Colgan et al., 1998[9]

Supplementary Table 3. Type specimen and n° of specimens examined with different methods in the presented study and in Worsaae et al., 2012. Abbreviations: α-tub, α-tubulin immunostaining (IS); CLSM, confocal laser scanning microscopy; FMRF, FMRF-amid-like IS; GLU, 2-3% glutaraldehyde; Hist, histological semithin sections examined in light microscopy; KW, Katrine Worsaae; PFA, 2-3% paraformaldehyde; phal, phalloidin; SEM, scanning electron microscopy; ser, serotonin IS; TB, toluidin blue staining of semithin sections on slide; TEM, transmission electron microscopy; tyr-tub, tyrosinated tubulin IS; UA + LC, uranyl-acetate + lead citrate staining of ultrathin sections on grid; WM, permanent whole mount (sealed, glycerol); WS, Wolfgang Sterrer.

**Species**	**Museum number**	**Coordinates**	**Storage medium**	**Fixation**	**Preparation**	**Staining**	**Sex**	**Depth (m)**	**Date (yyyy-mm-dd)**	**Legit**	**Paper**
M. psammophilus	NHMD-90314	16.8021 -88.0768	WM (holotype)	PFA	CLSM	tyr-tub, ser, dapi	male	14	2010-01-20	KW	Worsaae *et al.*, 2012[10]
	NHMD-90315	16.8021 -88.0768	WM	PFA	CLSM	α-tub, FMRF, dapi	male	14	2010-01-20	KW	Worsaae *et al.*, 2012[10]
	NHMD-90316	16.8021 -88.0768	WM	PFA	CLSM	tyr-tub, ser, phal, dapi	male	14	2010-01-20	KW	Worsaae *et al.*, 2012[10]
	NHMD-90317	16.8021 -88.0768	WM	PFA	CLSM	α-tub, FMRF, dapi	male	14	2010-01-20	KW	Worsaae *et al.*, 2012[10]
	NHMD-90318	16.8021 -88.0768	WM	PFA	CLSM	α-tub, FMRF, dapi	male	14	2010-01-20	KW	Worsaae *et al.*, 2012[10]
	NHMD-90319	16.8021 -88.0768	WM	PFA	CLSM	tyr-tub, ser, dapi	male	14	2010-01-20	KW	Worsaae *et al.*, 2012[10]
	NHMD-90320	16.8021 -88.0768	stub	GLU	SEM	none	-	14	2010-01-20	KW	Worsaae *et al.*, 2012[10]
	NHMD-90321	16.8021 -88.0768	stub	GLU	SEM	none	-	14	2010-01-20	KW	Worsaae *et al.*, 2012[10]
	NHMD-90322	16.8021 -88.0768	stub	GLU	SEM	none	-	14	2010-01-20	KW	Worsaae *et al.*, 2012[10]
	NHMD-90323	16.8021 -88.0768	stub	GLU	SEM	none	-	14	2010-01-20	KW	Worsaae *et al.*, 2012[10]
	NHMD-90324	16.8021 -88.0768	stub	GLU	SEM	none	-	14	2010-01-20	KW	Worsaae *et al.*, 2012[10]
	NHMD-90325	16.8021 -88.0768	stub	GLU	SEM	none	-	14	2010-01-20	KW	Worsaae *et al.*, 2012[10]
	NHMD-90326	16.8021 -88.0768	stub	GLU	SEM	none	-	14	2010-01-20	KW	Worsaae *et al.*, 2012[10]
	NHMD-90327	16.8021 -88.0768	stub	GLU	SEM	none	-	14	2010-01-20	KW	Worsaae *et al.*, 2012[10]
	NHMD-90328	16.8021 -88.0768	stub	GLU	SEM	none	-	14	2010-01-20	KW	Worsaae *et al.*, 2012[10]
	NHMD-90329	16.8021 -88.0768	stub	GLU	SEM	none	-	14	2010-01-20	KW	Worsaae *et al.*, 2012[10]
	NHMD-90330	16.8021 -88.0768	stub	GLU	SEM	none	-	14	2010-01-20	KW	Worsaae *et al.*, 2012[10]
	NHMD-90331	16.8021 -88.0768	resin	GLU	none	none	-	14	2010-01-20	KW	Worsaae *et al.*, 2012[10]
	NHMD-90332	16.8021 -88.0768	resin	GLU	none	none	-	14	2010-01-20	KW	Worsaae *et al.*, 2012[10]

	NHMD-90333	16.8021<br>-88.0768	resin	GLU	none	none	-	14	2010-01-20	KW	Worsaae *et al.*, 2012[10]
	NHMD-90334	16.8021<br>-88.0768	resin	GLU	none	none	-	14	2010-01-20	KW	Worsaae *et al.*, 2012[10]
	NHMD-90335	16.8021<br>-88.0768	resin	GLU	none	none	-	14	2010-01-20	KW	Worsaae *et al.*, 2012[10]
	NHMD-90336	16.8021<br>-88.0768	resin	GLU	none	none	-	14	2010-01-20	KW	Worsaae *et al.*, 2012[10]
	NHMD-90337	16.8021<br>-88.0768	resin	GLU	none	none	-	14	2010-01-20	KW	Worsaae *et al.*, 2012[10]
	NHMD-90338	16.8021<br>-88.0768	resin	GLU	none	none	-	14	2010-01-20	KW	Worsaae *et al.*, 2012[10]
	NHMD-90339	16.8021<br>-88.0768	resin	GLU	none	none	-	14	2010-01-20	KW	Worsaae *et al.*, 2012[10]
	NHMD-90340	16.8021<br>-88.0768	resin (slides)	GLU	Hist	TB	male	14	2010-01-20	KW	Worsaae *et al.*, 2012[10]
	NHMD-90341	16.8021<br>-88.0768	resin (slides)	GLU	Hist & TEM	TB or UA+LC	male	14	2010-01-20	KW	Worsaae *et al.*, 2012[10]
	NHMD-90342	16.8021<br>-88.0768	resin (grids and slides)	GLU	Hist	TB	male	14	2010-01-20	KW	Worsaae *et al.*, 2012[10]
	NHMD-90343	16.8021<br>-88.0768	resin (slides)	GLU	Hist & TEM	TB or UA+LC	male	14	2010-01-20	KW	Worsaae *et al.*, 2012[10]
	NHMD-90344	16.8021<br>-88.0768	resin (grids and slides)	GLU	Hist & TEM	TB or UA+LC	male	14	2010-01-20	KW	Worsaae *et al.*, 2012[10]
	NHMD-90345	16.8021<br>-88.0768	resin (grids and slides)	GLU	Hist & TEM	TB or UA+LC	male	14	2010-01-20	KW	Worsaae *et al.*, 2012[10]
	NHMD-90346	16.8021<br>-88.0768	resin (grids and slides)	GLU	Hist & TEM	TB or UA+LC	male	14	2010-01-20	KW	Worsaae *et al.*, 2012[10]
	NHMD-90347	16.8021<br>-88.0768	resin (grids and slides)	GLU	Hist & TEM	TB or UA+LC	male	14	2010-01-20	KW	Worsaae *et al.*, 2012[10]
	2 further specimens examinated	16.8021<br>-88.0768	grids lost	GLU	TEM	UA+LC	male	14	2010-01-20	KW	Worsaae *et al.*, 2012[10]
	NHMD-90349	16.8021<br>-88.0768	70% EtOH (ca. 1 specimen)	GLU	none	none	-	14	2010-01-20	KW	Worsaae *et al.*, 2012[10]
	NHMD-90350	16.8021<br>-88.0768	GLU (ca. 7 specimens)	GLU	none	none	-	14	2010-01-20	KW	Worsaae *et al.*, 2012[10]
	NHMD-90351	16.8021<br>-88.0768	PBS+NaN_3 (ca. 20 specimens)	PFA	none	none	-	14	2010-01-20	KW	Worsaae *et al.*, 2012[10]
	NHMD-90355	16.8021<br>-88.0768	96% EtOH (6 specimens)	96% EtOH	none	none	-	14	2010-01-20	KW	Worsaae *et al.*, 2012[10]
	NHMD-1731257	19.9829<br>-75.8665	WM	GLU	CLSM	tyr-tub, ser, dapi	male	15-17	2014-04-11	KW	Worsaae *et al.*, 2012[10]
M. bermudensis sp. nov.	NHMD-90356	32.3317<br>-64.6767	WM (holotype)	PFA	CLSM	α-tub, ser, phal, dapi	-	12	2007-10-18	KW& WS	Worsaae *et al.*, 2012[10]
	NHMD-90357	32.3317<br>-64.6767	WM	PFA	CLSM	α-tub, FMRF, dapi	-	12	2007-10-18	KW& WS	Worsaae *et al.*, 2012[10]
	NHMD-90358	32.3317<br>-64.6767	WM	PFA	CLSM	Phal, dapi	-	12	2007-10-18	KW& WS	Worsaae *et al.*, 2012[10]

NHMD-90359	32.3317 -64.6767	WM	PFA	CLSM	α-tub, FMRF, dapi	-	12	2007-10-18	KW& WS	Worsaae *et al.*, 2012[10]
NHMD-90360	32.3317 -64.6767	WM	PFA	CLSM	phal, dapi	-	12	2007-10-18	KW& WS	Worsaae *et al.*, 2012[10]
NHMD-90361	32.3317 -64.6767	WM	PFA	CLSM	α-tub, FMRF, dapi	-	12	2007-10-18	KW& WS	Worsaae *et al.*, 2012[10]
NHMD-90362	32.3317 -64.6767	WM	PFA	CLSM	α-tub, FMRF, dapi	-	12	2007-10-18	KW& WS	Worsaae *et al.*, 2012[10]
NHMD-90363	32.3317 -64.6767	WM	PFA	CLSM	α-tub, ser, phal, dapi	-	12	2007-10-18	KW& WS	Worsaae *et al.*, 2012[10]
NHMD-90364	32.3317 -64.6767	WM	PFA	CLSM	α-tub, ser, phal, dapi	-	12	2007-10-18	KW& WS	Worsaae *et al.*, 2012[10]
NHMD-90365	32.3317 -64.6767	WM	PFA	CLSM	α-tub, ser, phal, dapi	-	12	2007-10-18	KW& WS	Worsaae *et al.*, 2012[10]
NHMD-90366	32.3317 -64.6767	WM	PFA	CLSM	tyr-tub, ser, phal, dapi	-	12	2007-10-18	KW& WS	Worsaae *et al.*, 2012[10]
NHMD-90367	32.3317 -64.6767	WM	PFA	CLSM	tyr-tub, ser, phal, dapi	-	12	2007-10-18	KW& WS	Worsaae *et al.*, 2012[10]
NHMD-90368	32.3317 -64.6767	WM	PFA	CLSM	tyr-tub, ser, phal, dapi	-	12	2007-10-18	KW& WS	Worsaae *et al.*, 2012[10]
NHMD-90369	32.3317 -64.6767	WM	PFA	CLSM	tyr-tub, ser, phal, dapi	-	12	2007-10-18	KW& WS	Worsaae *et al.*, 2012[10]
NHMD-90370	32.3317 -64.6767	WM	PFA	CLSM	tyr-tub, ser, phal, dapi	-	12	2007-10-18	KW& WS	Worsaae *et al.*, 2012[10]
NHMD-90371	32.3317 -64.6767	WM	PFA	CLSM	tyr-tub, ser, phal, dapi	-	12	2007-10-18	KW& WS	Worsaae *et al.*, 2012[10]
NHMD-90372	32.3317 -64.6767	WM	PFA	CLSM	tyr-tub, ser, phal, dapi	-	12	2007-10-18	KW& WS	Worsaae *et al.*, 2012[10]
NHMD-90373	32.3317 -64.6767	WM	PFA	CLSM	α-tub, ser, phal, dapi	-	12	2007-10-18	KW& WS	Worsaae *et al.*, 2012[10]
NHMD-90374	32.3317 -64.6767	WM	PFA	CLSM	α-tub, ser, dapi	-	12	2007-10-18	KW& WS	Worsaae *et al.*, 2012[10]
NHMD-90375	32.3317 -64.6767	WM	PFA	CLSM	α-tub, ser, phal, dapi	-	12	2007-10-18	KW& WS	Worsaae *et al.*, 2012[10]
NHMD-90376	32.3317 -64.6767	WM	PFA	CLSM	α-tub, ser, phal, dapi	-	12	2007-10-18	KW& WS	Worsaae *et al.*, 2012[10]
NHMD-90377	32.3317 -64.6767	WM	PFA	CLSM	α-tub, ser, dapi	-	12	2007-10-18	KW& WS	Worsaae *et al.*, 2012[10]
NHMD-90378	32.3317 -64.6767	WM	PFA	CLSM	α-tub, FMRF, dapi	-	12	2007-10-18	KW& WS	Worsaae *et al.*, 2012[10]
NHMD-90379	32.3317 -64.6767	WM	PFA	CLSM	tyr-tub, phal, dapi	-	12	2007-10-18	KW& WS	Worsaae *et al.*, 2012[10]
NHMD-90380	32.3317 -64.6767	WM	PFA	CLSM	α-tub, FMRF dapi	-	12	2007-10-18	KW& WS	Worsaae *et al.*, 2012[10]
NHMD-90381	32.3317 -64.6767	stub	1% OsO_4	SEM	none	-	12	2007-10-18	KW& WS	Worsaae *et al.*, 2012[10]
NHMD-90382	32.3317 -64.6767	stub	1% OsO_4	SEM	none	-	12	2007-10-18	KW& WS	Worsaae *et al.*, 2012[10]

	NHMD-90383	32.3317 -64.6767	stub	1% OsO4	SEM	none	-	12	2007-10-18	KW& WS	Worsaae *et al.*, 2012[10]
	NHMD-90384	32.3317 -64.6767	stub	1% OsO4	SEM	none	-	12	2007-10-18	KW& WS	Worsaae *et al.*, 2012[10]
	NHMD-90385	32.3317 -64.6767	stub	1% OsO4	SEM	none	-	12	2007-10-18	KW& WS	Worsaae *et al.*, 2012[10]
	NHMD-90386	32.3317 -64.6767	PFA (ca, 4 specimens)	PFA	none	none	-	12	2007-10-18	KW& WS	Worsaae *et al.*, 2012[10]
	NHMD-90387	32.3317 -64.6767	PBS+NaN_3 (ca. 7 specimens)	PFA	none	none	-	12	2007-10-18	KW& WS	Worsaae *et al.*, 2012[10]
	NHMD-90388	32.3317 -64.6767	PBS+NaN_3 (ca. 5 specimens)	PFA	none	none	-	12	2007-10-18	KW& WS	Worsaae *et al.*, 2012[10]
	NHMD-90389	32.3317 -64.6767	Caco-buffer (10 specimens)	GLU	none	none	-	12	2007-10-18	KW& WS	Worsaae *et al.*, 2012[10]
	NHMD-90390	32.3317 -64.6767	Caco-buffer (7 specimens)	GLU	none	none	-	12	2007-10-18	KW& WS	Worsaae *et al.*, 2012[10]
	NHMD-90391	32.3317 -64.6767	Caco-buffer (5 specimens)	GLU	none	none	-	12	2007-10-18	KW& WS	Worsaae *et al.*, 2012[10]
	NHMD-90392	32.3317 -64.6767	96% EtOH (1 specimen)	96% EtOH	none	none	-	2-4	2007-10-18	KW& WS	Worsaae *et al.*, 2012[10]
	NHMD-90393	32.3317 -64.6767	96% EtOH (5 specimens)	96% EtOH	none	none	-	12	2007-10-18	KW& WS	Worsaae *et al.*, 2012[10]
	NHMD-90394	32.3317 -64.6767	96% EtOH (1 specimen)	96% EtOH	none	none	-	12	2007-10-18	KW& WS	Worsaae *et al.*, 2012[10]
	NHMD-90395	32.3317 -64.6767	96% EtOH (1 specimen)	96% EtOH	none	none	-	12	2007-10-18	KW& WS	Worsaae *et al.*, 2012[10]
	NHMD-90396	32.3317 -64.6767	96% EtOH (1 specimen)	96% EtOH	none	none	-	12	2007-10-18	KW& WS	Worsaae *et al.*, 2012[10]
M. chiviricoensis sp. nov.	NHMD-90348	16.8037 -88.0769	GLU (ca. 5 specimens)	GLU	none	none	-	15	2010-01-16	KW	Worsaae *et al.*, 2012[10]
	NHMD-90352	16.8037 -88.0769	96% EtOH (4 specimens)	96% EtOH	none	none	-	15	2010-01-16	KW	Worsaae *et al.*, 2012[10]
	NHMD-90353	16.8037 -88.0769	96% EtOH (6 specimens)	96% EtOH	none	none	-	15	2010-01-16	KW	Worsaae *et al.*, 2012[10]
	NHMD-90354	16.8037 -88.0769	96% EtOH (5 specimens)	96% EtOH	none	none	-	15	2010-01-16	KW	Worsaae *et al.*, 2012[10]
	NHMD-1731258	19.9686 -76.4084	WM	PFA	CLSM	tyr-tub, ser, dapi	male	29	2014-11-23	KW	This paper
	NHMD-1731259	19.9686 -76.4084	WM (holotype)	PFA	CLSM	α tub, FMRF, dapi	male	29	2014-11-23	KW	This paper
	NHMD-1731260	19.9686 -76.4084	WM	PFA	CLSM	α tub, FMRF, dapi	male	29	2014-11-23	KW	This paper
	NHMD-1731261	19.9686 -76.4084	WM	PFA	CLSM	tyr-tub, ser, dapi	-	29	2014-11-23	KW	This paper
	NHMD-1731262	19.9686 -76.4084	WM	PFA	CLSM	tyr-tub, ser, dapi	-	29	2014-11-23	KW	This paper
M. curacaoensis sp.nov.	NHMD-1731263	12.1479 -68.9990	96% EtOH (holotype)	96% EtOH	none	none	-	0.3	2018-04-21	MH	This paper
	NHMD-1731264	12.1479	96% EtOH	96% EtOH	none	none	-	0.3	2018-04-21	MH	This paper

		-68.9990									
	NHMD-1731265	12.1479<br>-68.9990	96% EtOH	96% EtOH	none	none	-	0.3	2018-04-21	MH	This paper
M. eilatensis sp.nov	NHMD-1731266	29.5164<br>34.9284	96% EtOH (holotype)	96% EtOH	none	none	-	15-18	2014-02-11	KW	This paper
M. iuiensis sp. nov.	NHMD-1731266	29.5164<br>34.9169	WM (holotype)	96% EtOH	CLSM	tyr-tub, ser, dapi	-	8	2014-02-18	KW	This paper
	NHMD-1731268	29.5164<br>34.9169	WM	96% EtOH	CLSM	tyr-tub, ser, dapi	-	8	2014-02-18	KW	This paper
M. jejuensis sp. nov.	NHMD-1731269	33.2294<br>126.6027	WM<br>(holotype)	PFA	CLSM	tyr-tub, ser, dapi	-	33	2015-05-19	KW	This paper
	NIBRIV0000910960	33.2294<br>126.6027	96% EtOH	PFA	none	none	-	15	2015-05-25	KW	This paper
	NHMD-1731270	33.2294<br>126.6027	96% EtOH	3% GLU	none	none	-	33	2015-05-19	KW	This paper
	NHMD-1731271	33.2304<br>126.6027	96% EtOH	3% GLU	none	none	-	14	2018-05-28	KW	This paper
	NHMD-1731272	33.2304<br>126.6027	96% EtOH	3% GLU	none	none	-	14	2018-05-28	KW	This paper
	NHMD-1731273	33.2304<br>126.6027	96% EtOH	3% GLU	none	none	-	14	2018-05-28	KW	This paper
M. maldivensis sp.<br>nov.	NHMD-1731274	0.1993<br>73.2204	96% EtOH (holotype)	96% EtOH	none	none	-	10-14	2021-11-22	KW	This paper
	NHMD-1731275	0.2275<br>73.2131	WM	PFA	CLSM	tyr-tub, ser, dapi	-	12.3	2021-11-30	KW	This paper
M. turkensis sp. nov.	NHMD-1731276	21.7898<br>-72.2273	96% EtOH (holotype)	PFA	none	none	-	1.5	2019-01-06	KW	This paper
	NHMD-1731277	21.7898<br>-72.2273	96% EtOH	PFA	one	none	-	1.5	2019-01-06	KW	This paper

Supplementary Table 4. Similarity matrix using 16S rRNA dataset (values are given in %). See Suppl. Table 1 for ID of species.

	Belize KW096 B	Chivi. KW702	Chivi. KW785	Chivi. KW786	Berm. KWB	Belize KW099 A	Belize KW099 B	Belize KW099 C	Belize KW099 D	Belize KW099 E	Perdiz KW511	Perdiz KW546	Mira. KW512	Mira. KW802	Cura. KW795	Cura. KW796	Cura. KW797	Cura. KW798	Cura. KW799	Giba. KW700	Giba. KW792	T & C KW803	T & C KW804	Eilat 32 KW513	Eilat 32 KW791	Eilat 32 KW790	Maldi KW878	Maldi KW881	Maldi KW882	Maldi KW883	S. Kor KW718	S. Kor KW793	S. Kor KW788	S. Kor KW789	S. Kor KW794	Eilat 16 KW800	Eilat 16 KW801
Belize KW096 B		99.49	99.49	99.49	83.98	80.03	80.03	80.03	80.03	80.03	80.34	80.34	80.59	80.67	81.01	81.01	81.01	81.01	81.01	80.92	81.01	80.87	80.87	82.40	82.40	82.40	81.94	81.73	81.73	81.73	81.01	81.01	81.01	81.01	81.01	81.14	81.14
Chivi. KW702	99.49		100	100	83.89	80.20	80.20	80.20	80.20	80.20	80.89	80.89	81.14	81.22	81.22	81.22	81.22	81.22	81.22	81.14	81.22	81.09	81.09	82.75	82.75	82.72	82.40	82.09	82.09	82.09	81.05	81.05	81.05	81.05	81.05	81.35	81.35
Chivi. KW785	99.49	100		100	83.89	80.20	80.20	80.20	80.20	80.20	80.89	80.89	81.14	81.22	81.22	81.22	81.22	81.22	81.22	81.14	81.22	81.09	81.09	82.75	82.75	82.72	82.40	82.09	82.09	82.09	81.05	81.05	81.05	81.05	81.05	81.35	81.35
Chivi. KW786	99.49	100	100		83.89	80.20	80.20	80.20	80.20	80.20	80.89	80.89	81.14	81.22	81.22	81.22	81.22	81.22	81.22	81.14	81.22	81.09	81.09	82.75	82.75	82.72	82.40	82.09	82.09	82.09	81.05	81.05	81.05	81.05	81.05	81.35	81.35
Berm. KWB	83.98	83.89	83.89	83.89		80.44	80.44	80.44	80.44	80.44	80.84	80.84	80.62	80.70	81.18	81.18	81.18	81.18	81.18	81.18	81.34	81.21	81.21	83.25	83.25	83.25	83.38	83.11	83.11	83.11	82.35	82.35	82.35	82.35	82.35	80.61	80.61
Belize KW099 A	80.03	80.20	80.20	80.20	80.44		100	100	100	100	99.66	99.66	98.90	98.98	96.44	96.44	96.44	96.44	96.44	95.59	95.76	95.93	95.93	85.76	85.76	85.76	86.09	85.93	85.93	85.93	85.02	85.02	85.02	85.02	85.02	84.63	84.63
Belize KW099 B	80.03	80.20	80.20	80.20	80.44	100		100	100	100	99.66	99.66	98.90	98.98	96.44	96.44	96.44	96.44	96.44	95.59	95.76	95.93	95.93	85.76	85.76	85.76	86.09	85.93	85.93	85.93	85.02	85.02	85.02	85.02	85.02	84.63	84.63
Belize KW099 C	80.03	80.20	80.20	80.20	80.44	100	100		100	100	99.66	99.66	98.90	98.98	96.44	96.44	96.44	96.44	96.44	95.59	95.76	95.93	95.93	85.76	85.76	85.76	86.09	85.93	85.93	85.93	85.02	85.02	85.02	85.02	85.02	84.63	84.63
Belize KW099 D	80.03	80.20	80.20	80.20	80.44	100	100	100		100	99.66	99.66	98.90	98.98	96.44	96.44	96.44	96.44	96.44	95.59	95.76	95.93	95.93	85.76	85.76	85.76	86.09	85.93	85.93	85.93	85.02	85.02	85.02	85.02	85.02	84.63	84.63
Belize KW099 E	80.03	80.20	80.20	80.20	80.44	100	100	100	100		99.66	99.66	98.90	98.98	96.44	96.44	96.44	96.44	96.44	95.59	95.76	95.93	95.93	85.76	85.76	85.76	86.09	85.93	85.93	85.93	85.02	85.02	85.02	85.02	85.02	84.63	84.63
Perdiz KW511	80.34	80.89	80.89	80.89	80.84	99.66	99.66	99.66	99.66	99.66		100	99.25	99.34	96.84	96.84	96.84	96.84	96.84	96.01	96.17	96.01	96.01	86.36	86.36	86.33	86.63	86.36	86.36	86.36	85.62	85.62	85.62	85.62	85.62	85.24	85.24
Perdiz KW546	80.34	80.89	80.89	80.89	80.84	99.66	99.66	99.66	99.66	99.66	100		99.25	99.34	96.84	96.84	96.84	96.84	96.84	96.01	96.17	96.01	96.01	86.36	86.36	86.33	86.63	86.36	86.36	86.36	85.62	85.62	85.62	85.62	85.62	85.24	85.24
Mira. KW512	80.59	81.14	81.14	81.14	80.62	98.90	98.90	98.90	98.90	98.90	99.25	99.25		99.92	96.76	96.76	96.76	96.76	96.76	96.01	96.10	95.94	95.94	86.30	86.30	86.27	86.57	86.30	86.30	86.30	85.23	85.23	85.23	85.23	85.23	85.18	85.18
Mira. KW802	80.67	81.22	81.22	81.22	80.70	98.98	98.98	98.98	98.98	98.98	99.34	99.34	99.92		96.84	96.84	96.84	96.84	96.84	96.01	96.18	96.02	96.02	86.38	86.38	86.36	86.65	86.38	86.38	86.38	85.31	85.31	85.31	85.31	85.31	85.26	85.26
Cura. KW795	81.01	81.22	81.22	81.22	81.18	96.44	96.44	96.44	96.44	96.44	96.84	96.84	96.76	96.84		100	100	100	100	96.51	96.67	96.51	96.51	87.02	87.02	87.00	86.98	86.69	86.69	86.69	85.62	85.62	85.62	85.62	85.62	85.07	85.07
Cura. KW796	81.01	81.22	81.22	81.22	81.18	96.44	96.44	96.44	96.44	96.44	96.84	96.84	96.76	96.84	100		100	100	100	96.51	96.67	96.51	96.51	87.02	87.02	87.00	86.98	86.69	86.69	86.69	85.62	85.62	85.62	85.62	85.62	85.07	85.07
Cura. KW797	81.01	81.22	81.22	81.22	81.18	96.44	96.44	96.44	96.44	96.44	96.84	96.84	96.76	96.84	100	100		100	100	96.51	96.67	96.51	96.51	87.02	87.02	87.00	86.98	86.69	86.69	86.69	85.62	85.62	85.62	85.62	85.62	85.07	85.07
Cura. KW798	81.01	81.22	81.22	81.22	81.18	96.44	96.44	96.44	96.44	96.44	96.84	96.84	96.76	96.84	100	100	100		100	96.51	96.67	96.51	96.51	87.02	87.02	87.00	86.98	86.69	86.69	86.69	85.62	85.62	85.62	85.62	85.62	85.07	85.07
Cura. KW799	81.01	81.22	81.22	81.22	81.18	96.44	96.44	96.44	96.44	96.44	96.84	96.84	96.76	96.84	100	100	100	100		96.51	96.67	96.51	96.51	87.02	87.02	87.00	86.98	86.69	86.69	86.69	85.62	85.62	85.62	85.62	85.62	85.07	85.07
Gibara KW700	80.92	81.14	81.14	81.14	81.18	95.59	95.59	95.59	95.59	95.59	96.01	96.01	96.01	96.01	96.51	96.51	96.51	96.51	96.51		99.83	99.67	99.67	86.52	86.52	86.50	86.80	86.52	86.52	86.52	85.29	85.29	85.29	85.29	85.29	85.49	85.49
Gibara KW792	81.01	81.22	81.22	81.22	81.34	95.76	95.76	95.76	95.76	95.76	96.17	96.17	96.10	96.18	96.67	96.67	96.67	96.67	96.67	99.83		99.83	99.83	86.69	86.69	86.67	86.98	86.69	86.69	86.69	85.45	85.45	85.45	85.45	85.45	85.57	85.57
T & C KW803	80.87	81.09	81.09	81.09	81.21	95.93	95.93	95.93	95.93	95.93	96.01	96.01	95.94	96.02	96.51	96.51	96.51	96.51	96.51	99.67	99.83		100	86.54	86.54	86.52	86.83	86.54	86.54	86.54	85.31	85.31	85.31	85.31	85.31	85.43	85.43
T & C KW804	80.87	81.09	81.09	81.09	81.21	95.93	95.93	95.93	95.93	95.93	96.01	96.01	95.94	96.02	96.51	96.51	96.51	96.51	96.51	99.67	99.83	100		86.54	86.54	86.52	86.83	86.54	86.54	86.54	85.31	85.31	85.31	85.31	85.31	85.43	85.43
Eilat 32 KW513	82.40	82.75	82.75	82.75	83.25	85.76	85.76	85.76	85.76	85.76	86.36	86.36	86.30	86.38	87.02	87.02	87.02	87.02	87.02	86.52	86.69	86.54	86.54		100	100	96.89	96.98	96.98	96.98	87.85	87.85	87.85	87.85	87.85	86.33	86.33
Eilat 32 KW591	82.40	82.75	82.75	82.75	83.25	85.76	85.76	85.76	85.76	85.76	86.36	86.36	86.30	86.38	87.02	87.02	87.02	87.02	87.02	86.52	86.69	86.54	86.54	100		100	96.89	96.98	96.98	96.98	87.85	87.85	87.85	87.85	87.85	86.33	86.33
Eilat 32 KW590	82.40	82.72	82.72	82.72	83.25	85.76	85.76	85.76	85.76	85.76	86.33	86.33	86.27	86.36	87.00	87.00	87.00	87.00	87.00	86.50	86.67	86.52	86.52	100	100		96.89	96.97	96.97	96.97	87.83	87.83	87.83	87.83	87.83	86.31	86.31
Maldi. KW878	81.94	82.40	82.40	82.40	83.38	86.09	86.09	86.09	86.09	86.09	86.63	86.63	86.57	86.65	86.98	86.98	86.98	86.98	86.98	86.80	86.98	86.83	86.83	96.89	96.89	96.89		99.69	99.69	99.69	88.04	88.04	88.04	88.04	88.04	87.00	87.00
Maldi. KW881	81.73	82.09	82.09	82.09	83.11	85.93	85.93	85.93	85.93	85.93	86.36	86.36	86.30	86.38	86.69	86.69	86.69	86.69	86.69	86.52	86.69	86.54	86.54	96.98	96.98	96.97	99.69		100	100	88.00	88.00	88.00	88.00	88.00	86.83	86.83
Maldi. KW882	81.73	82.09	82.09	82.09	83.11	85.93	85.93	85.93	85.93	85.93	86.36	86.36	86.30	86.38	86.69	86.69	86.69	86.69	86.69	86.52	86.69	86.54	86.54	96.98	96.98	96.97	99.69	100		100	88.00	88.00	88.00	88.00	88.00	86.83	86.83
Maldi. KW883	81.73	82.09	82.09	82.09	83.11	85.93	85.93	85.93	85.93	85.93	86.36	86.36	86.30	86.38	86.69	86.69	86.69	86.69	86.69	86.52	86.69	86.54	86.54	96.98	96.98	96.97	99.69	100	100		88.00	88.00	88.00	88.00	88.00	86.83	86.83
S. Kor. KW718	81.01	81.05	81.05	81.05	82.35	85.02	85.02	85.02	85.02	85.02	85.62	85.62	85.23	85.31	85.62	85.62	85.62	85.62	85.62	85.29	85.45	85.31	85.31	87.85	87.85	87.83	88.04	88.00	88.00	88.00		100	100	100	100	87.58	87.58

S. Kor. KW793	81.01	81.05	81.05	81.05	82.35	85.02	85.02	85.02	85.02	85.02	85.62	85.62	85.23	85.31	85.62	85.62	85.62	85.62	85.62	85.29	85.45	85.31	85.31	87.85	87.85	87.83	88.04	88.00	88.00	88.00	100		100	100	100	87.58	87.58
S. Kor. KW788	81.01	81.05	81.05	81.05	82.35	85.02	85.02	85.02	85.02	85.02	85.62	85.62	85.23	85.31	85.62	85.62	85.62	85.62	85.62	85.29	85.45	85.31	85.31	87.85	87.85	87.83	88.04	88.00	88.00	88.00	100	100		100	100	87.58	87.58
S. Kor. KW789	81.01	81.05	81.05	81.05	82.35	85.02	85.02	85.02	85.02	85.02	85.62	85.62	85.23	85.31	85.62	85.62	85.62	85.62	85.62	85.29	85.45	85.31	85.31	87.85	87.85	87.83	88.04	88.00	88.00	88.00	100	100	100		100	87.58	87.58
S. Kor. KW794	81.01	81.05	81.05	81.05	82.35	85.02	85.02	85.02	85.02	85.02	85.62	85.62	85.23	85.31	85.62	85.62	85.62	85.62	85.62	85.29	85.45	85.31	85.31	87.85	87.85	87.83	88.04	88.00	88.00	88.00	100	100	100	100		87.58	87.58
Eilat 16 KW800	81.14	81.35	81.35	81.35	80.61	84.63	84.63	84.63	84.63	84.63	85.24	85.24	85.18	85.26	85.07	85.07	85.07	85.07	85.07	85.49	85.57	85.43	85.43	86.33	86.33	86.31	87.00	86.83	86.83	86.83	87.58	87.58	87.58	87.58	87.58		100
Eilat 16 KW801	81.14	81.35	81.35	81.35	80.61	84.63	84.63	84.63	84.63	84.63	85.24	85.24	85.18	85.26	85.07	85.07	85.07	85.07	85.07	85.49	85.57	85.43	85.43	86.33	86.33	86.31	87.00	86.83	86.83	86.83	87.58	87.58	87.58	87.58	87.58	100	

Supplementary Table 5. Similarity matrix using COI dataset (values are given in %). See Suppl. Table 1 for ID of species.

	Belize KW096 B	Chivi. KW702	Chivi. KW785	Chivi. KW786	Berm. KWB	Eilat 32 KW513	Eilat 32 KW791	Eilat 32 KW790	Maldi. KW881	Maldi. KW882	Maldi. KW883	S. Kor. KW718	S. Kor. KW788	S. Kor. KW789	S. Kor. KW794	S. Kor. KW793	Perdiz KW546	Gibara KW700	Gibara KW792	T & C KW803	T & C KW804	Cura. KW795	Cura. KW797	Cura. KW798	Cura. KW799	Mira. KW802	Eilat 16 KW801
Belize KW096 B		99.85	99.85	99.85	88.99	87.69	87.61	87.46	87.91	87.91	87.69	84.22	85.78	85.78	85.78	85.78	86.87	86.66	86.85	86.85	86.85	87.31	87.31	87.31	87.31	87.31	86.07
Chivi. KW702	99.85		100	100	88.84	87.89	87.65	87.67	87.91	87.91	87.63	84.21	85.90	85.99	86.05	85.76	86.47	86.70	87.22	87.08	87.08	87.56	87.67	87.52	87.52	87.61	85.55
Chivi. KW785	99.85	100		100	88.84	87.89	87.65	87.67	87.91	87.91	87.63	84.21	85.90	85.99	86.05	85.76	86.47	86.70	87.22	87.08	87.08	87.56	87.67	87.52	87.52	87.61	85.55
Chivi. KW786	99.85	100	100		88.84	87.83	87.65	87.61	87.91	87.91	87.72	84.13	85.99	85.99	85.99	85.99	86.73	86.79	87.17	87.17	87.17	87.56	87.61	87.61	87.61	87.61	85.48
Berm. KWB	88.99	88.84	88.84	88.84		87.65	87.65	87.50	87.20	87.20	87.31	83.88	85.57	85.57	85.57	85.57	88.11	87.88	87.95	87.95	87.95	87.80	87.80	87.80	87.80	88.54	86.30
Eilat 32 KW513	87.69	87.89	87.89	87.83	87.65		99.55	99.41	98.01	98.01	97.78	86.57	88.18	88.27	88.33	88.03	88.70	88.63	89.13	89.13	89.13	89.63	89.72	89.57	89.57	89.23	87.44
Eilat 32 KW791	87.61	87.65	87.65	87.65	87.65	99.55		99.48	98.07	98.07	97.97	86.36	88.24	88.24	88.24	88.24	89.20	88.78	89.14	89.29	89.29	89.73	89.73	89.73	89.73	89.29	87.74
Eilat 32 KW790	87.46	87.67	87.67	87.61	87.50	99.41	99.48		97.79	97.79	97.56	86.40	88.03	88.13	88.18	87.89	88.53	88.48	88.99	88.99	88.99	89.48	89.57	89.43	89.43	89.09	87.28
Maldi. KW881	87.77	87.91	87.91	87.91	87.20	98.01	98.07	97.79		100	99.93	87.35	88.79	88.79	88.79	88.79	88.97	88.58	88.94	89.09	89.09	89.78	89.82	89.82	89.82	89.38	87.86
Maldi. KW882	87.77	87.91	87.91	87.91	87.20	98.01	98.07	97.79	100		99.93	87.35	88.79	88.79	88.79	88.79	88.97	88.58	88.94	89.09	89.09	89.78	89.82	89.82	89.82	89.38	87.86
Maldi. KW883	87.69	87.63	87.63	87.72	87.31	97.78	97.97	97.56	99.93	99.93		87.35	88.67	88.62	88.52	88.52	88.78	88.56	88.67	88.96	88.96	89.61	89.56	89.70	89.70	89.21	87.86
S. Kor. KW718	84.22	84.21	84.21	84.13	83.88	86.57	86.36	86.40	87.35	87.35	87.16		98.56	98.73	98.73	98.40	84.71	85.39	85.56	85.56	85.56	85.07	85.22	85.05	85.05	84.97	86.15
S. Kor. KW788	85.78	85.90	85.90	85.99	85.57	88.18	88.24	88.03	88.79	88.79	88.67	98.56		100	99.85	99.85	85.97	87.30	87.37	87.67	87.67	87.26	87.22	87.37	87.37	87.02	87.28
S. Kor. KW789	85.78	85.99	85.99	85.99	85.57	88.27	88.24	88.13	88.79	88.79	88.62	98.73	100		100	100	86.07	87.24	87.46	87.61	87.61	87.26	87.32	87.32	87.32	87.02	87.38
S. Kor. KW794	85.78	86.05	86.05	85.99	85.57	88.33	88.24	88.18	88.79	88.79	88.52	98.73	99.85	100		99.71	85.81	87.15	87.52	87.52	87.52	87.26	87.37	87.22	87.22	87.02	87.44
S. Kor. KW793	85.78	85.76	85.76	85.99	85.57	88.03	88.24	87.89	88.79	88.79	88.52	98.40	99.85	100	99.71		86.14	87.15	87.22	87.52	87.52	87.26	87.08	87.22	87.22	87.02	87.12
Perdiz KW546	86.87	86.47	86.47	86.73	88.11	88.70	89.20	88.53	88.97	88.97	88.78	84.71	85.97	86.07	85.81	86.14		96.53	96.37	96.53	96.53	96.92	96.45	96.62	96.62	99.00	85.15
Gibara KW700	86.66	86.70	86.70	86.79	87.88	88.63	88.78	88.48	88.58	88.58	88.56	85.39	87.30	87.24	87.15	87.15	96.53		99.59	99.44	99.44	97.05	96.93	97.07	97.07	97.06	86.37
Gibara KW792	86.85	87.22	87.22	87.17	87.95	89.13	89.14	88.99	88.94	88.94	88.67	85.56	87.37	87.46	87.52	87.22	96.37	99.59		99.56	99.56	97.33	97.36	97.21	97.21	97.35	86.65
T & C KW803	86.85	87.08	87.08	87.17	87.95	89.13	89.29	88.99	89.09	89.09	88.96	85.56	87.67	87.61	87.52	87.52	96.53	99.44	99.56		100	97.04	96.92	97.06	97.06	97.35	86.65
T & C KW804	86.85	87.08	87.08	87.17	87.95	89.13	89.29	88.99	89.09	89.09	88.96	85.56	87.67	87.61	87.52	87.52	96.53	99.44	99.56	100		97.04	96.92	97.06	97.06	97.35	86.65
Curaçao KW795	87.31	87.56	87.56	87.56	87.80	89.63	89.73	89.48	89.78	89.78	89.61	85.07	87.26	87.26	87.26	87.26	96.92	96.77	97.33	97.04	97.04		100	100	100	97.33	87.32
Cura. KW797	87.31	87.67	87.67	87.61	87.80	89.72	89.73	89.57	89.82	89.82	89.56	85.22	87.22	87.32	87.37	87.08	96.45	96.64	97.36	96.92	96.92	100		99.85	99.85	97.35	87.12
Curaç. KW798	87.31	87.52	87.52	87.61	87.80	89.57	89.73	89.43	89.82	89.82	89.70	85.05	87.37	87.32	87.22	87.22	96.62	96.79	97.21	97.06	97.06	100	99.85		100	97.35	86.97
Cura. KW799	87.31	87.52	87.52	87.61	87.80	89.57	89.73	89.43	89.82	89.82	89.70	85.05	87.37	87.32	87.22	87.22	96.62	96.79	97.21	97.06	97.06	100	99.85	100		97.35	86.97
Mira. KW802	87.31	87.61	87.61	87.61	88.54	89.23	89.29	89.09	89.38	89.38	89.21	84.97	87.02	87.02	87.02	87.02	99.00	96.06	97.35	97.35	97.35	97.33	97.35	97.35	97.35		85.79
Eilat 16 KW801	86.07	85.55	85.55	85.48	86.30	87.44	87.74	87.28	87.86	87.86	87.86	86.15	87.28	87.38	87.44	87.12	85.15	86.37	86.65	86.65	86.65	87.32	87.12	86.97	86.97	85.79	

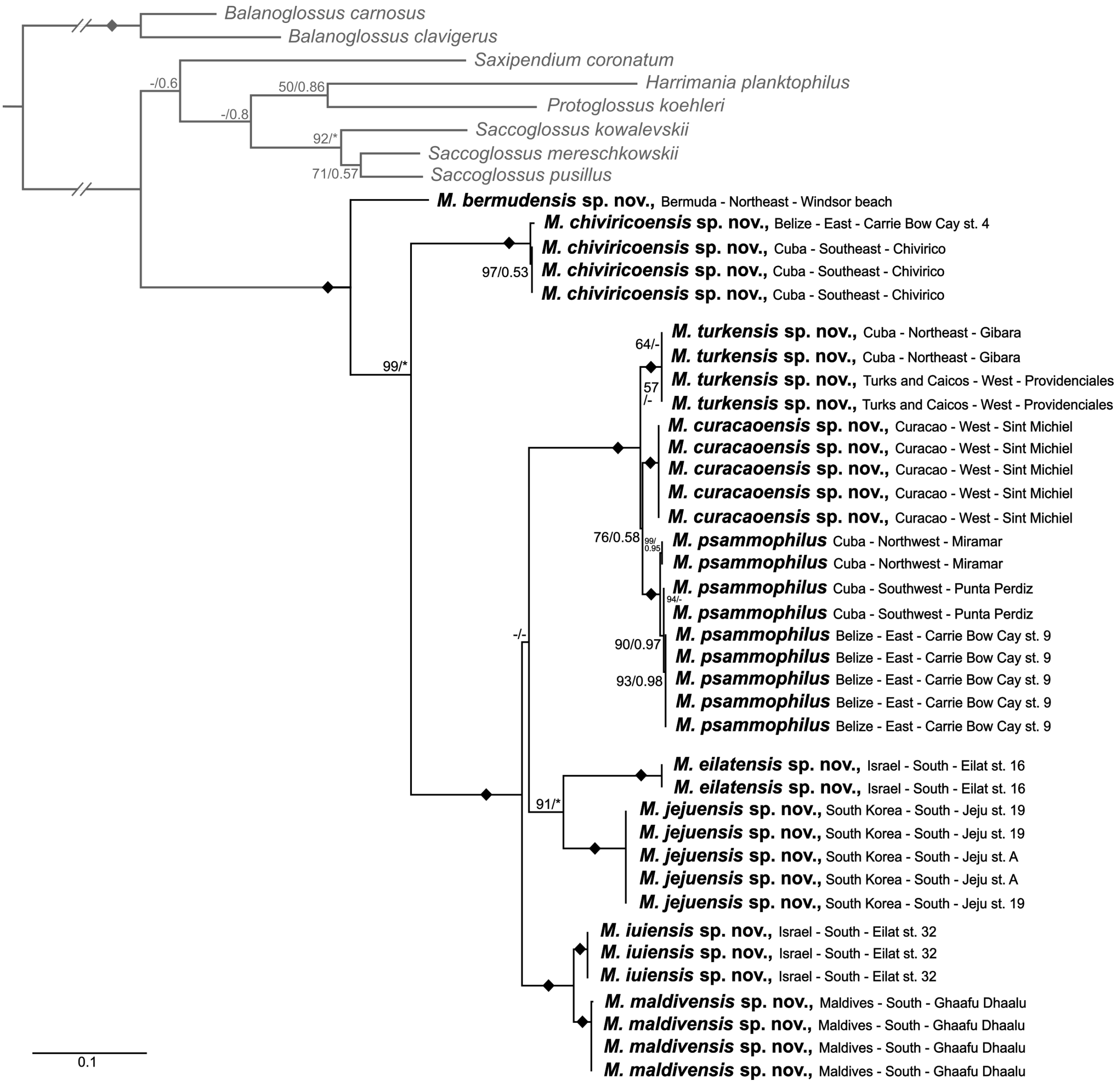

Supplementary Figure 1. Single gene tree. Phylogenetic relationship of *Meioglossus*, using 16S rRNA dataset. Topology based on Maximum Likelihood (LM) analysis. Nodal support is indicated with both Maximum Likelihood Bootstrapping (BS) and Bayesian Posterior Probabilities of the consensus tree (PP). Only nodal support above BS > 50% or PP > 0.5 are shown. Those falling below this threshold are represented by a dash (-). Asterisks indicate maximum support in either BS = 100% or PP = 1. Diamond (◆) shapes indicates full support in both analyses.

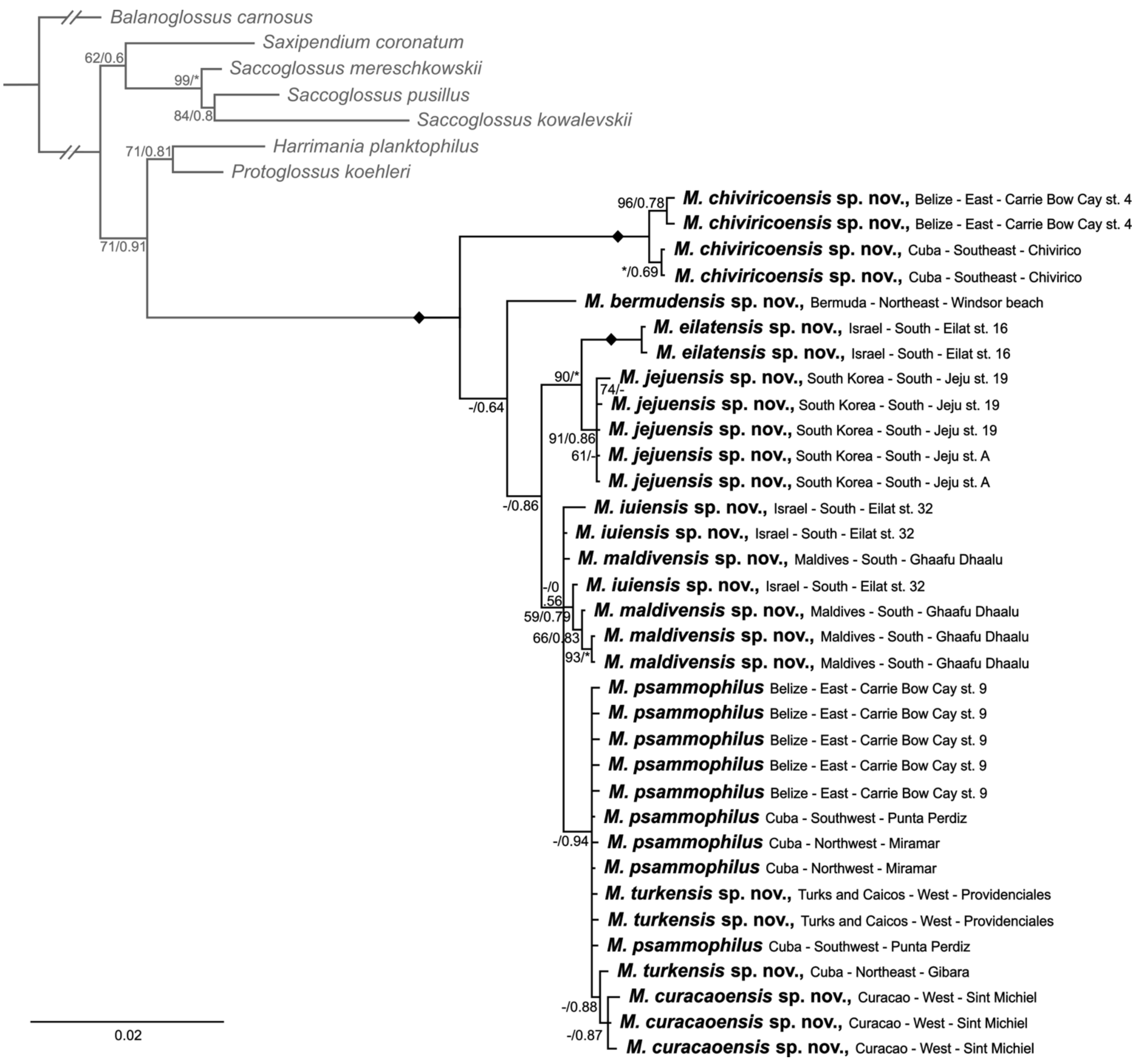

Supplementary Figure 2. Single gene tree. Phylogenetic relationship of *Meioglossus*, using 18S rRNA dataset. Topology based on Bayesian Inference (BI) analysis. Nodal support is indicated with both Maximum Likelihood Bootstrapping (BS) and Bayesian Posterior Probabilities of the consensus tree (PP). Only nodal support above BS > 50% or PP > 0.5 are shown. Those falling below this threshold are represented by a dash (-). Asterisks indicate maximum support in either BS = 100% or PP = 1. Diamond (◆) shapes indicates full support in both analyses.

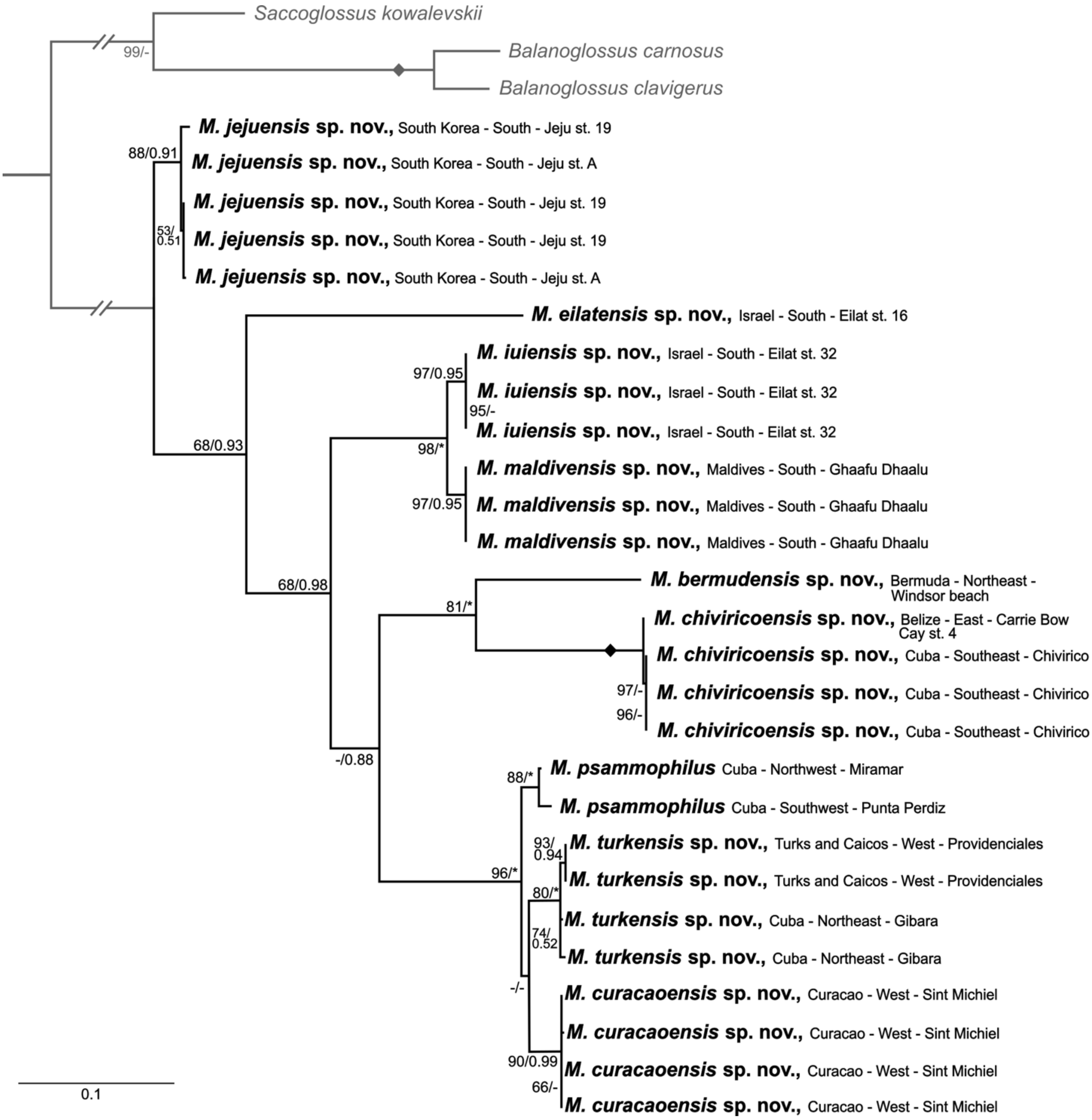

Supplementary Figure 3. Single gene tree. Phylogenetic relationship of *Meioglossus*, using COI dataset. Topology based on Maximum Likelihood (LM) analysis. Nodal support is indicated with both Maximum Likelihood Bootstrapping (BS) and Bayesian Posterior Probabilities of the consensus tree (PP). Only nodal support above BS > 50% or PP > 0.5 are shown. Those falling below this threshold are represented by a dash (-). Asterisks indicate maximum support in either BS = 100% or PP = 1. Diamond (◆) shapes indicates full support in both analyses.

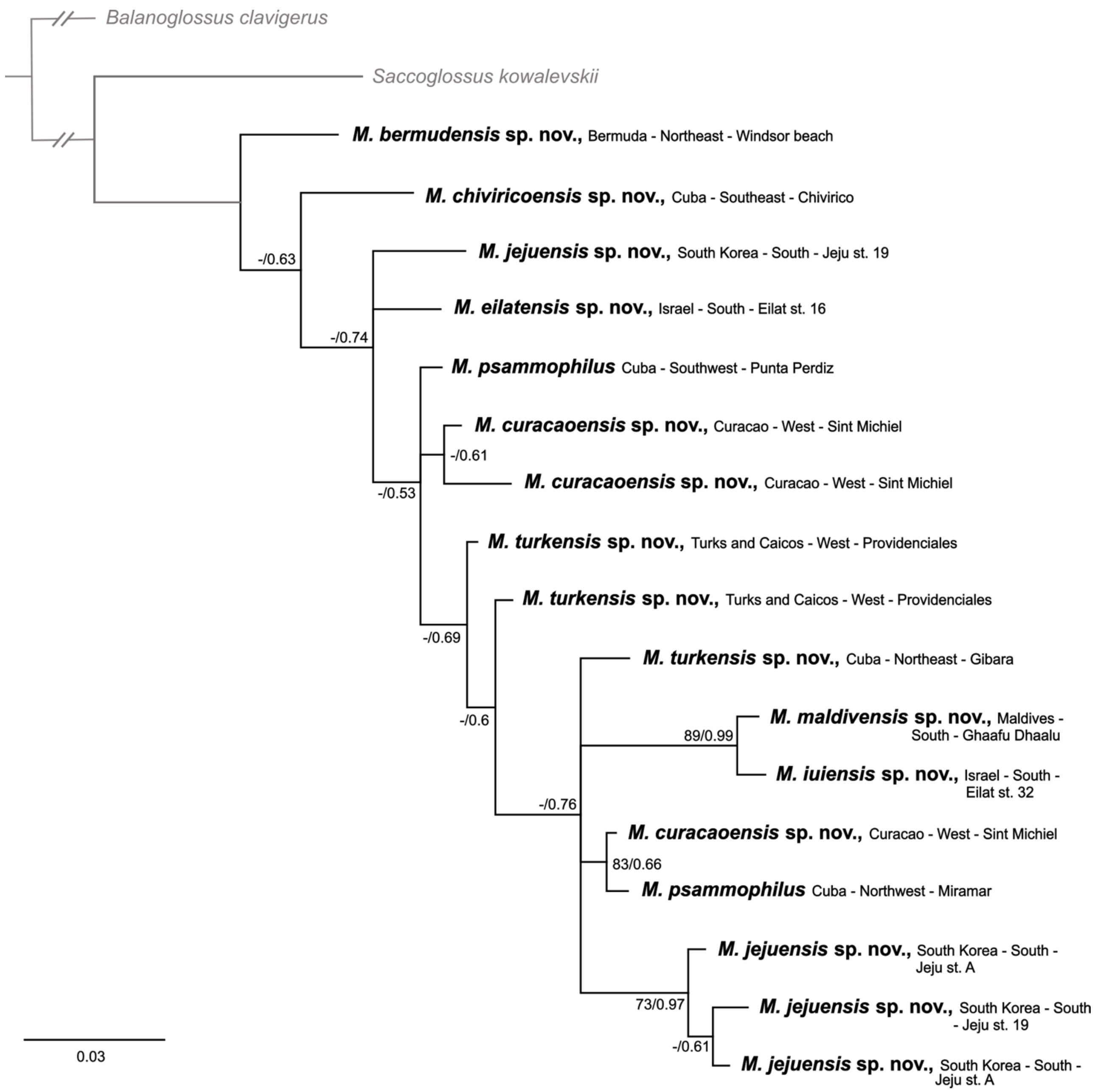

Supplementary Figure 4. Single gene tree. Phylogenetic relationship of *Meioglossus*, using H3 dataset. Topology based on Bayesian Inference (BI) analysis. Nodal support is indicated with both Maximum Likelihood Bootstrapping (BS) and Bayesian Posterior Probabilities of the consensus tree (PP). Only nodal support above BS > 50% or PP > 0.5 are shown. Those falling below this threshold are represented by a dash (-).

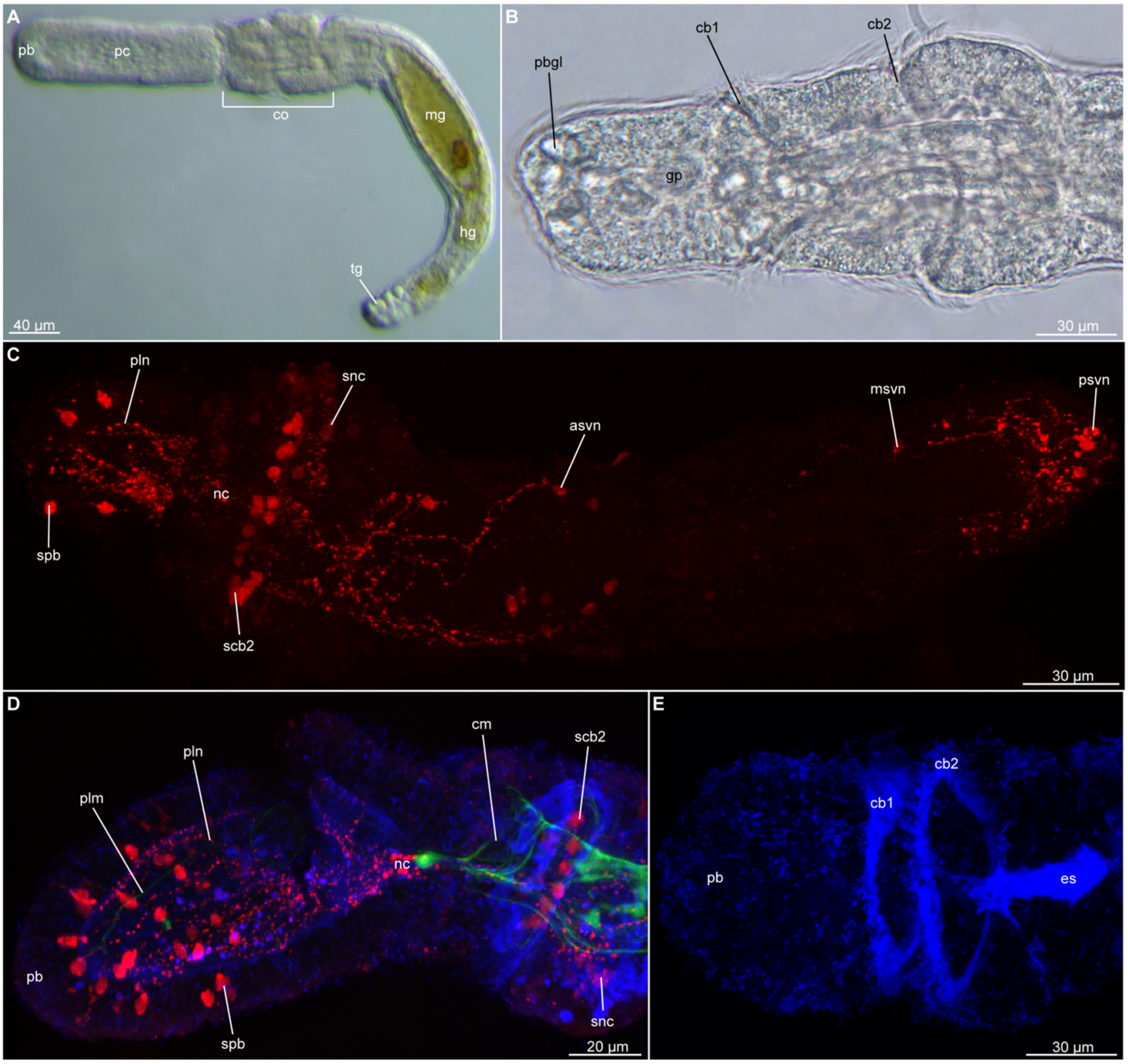

Supplementary Figure 5. *Meioglossus bermudensis* sp. nov., light- and confocal microscopy (LM and CLSM). A. Overview of *M. bermudensis* sp. nov., lateral view, dissecting scope. B. Close up of *M. bermudensis* sp. nov. anterior part, dorsal view, LM. C. Whole specimen, single staining, Serotonin-LIR (red), lateral view, CLSM (NHMD-90365). D. Close up of *M. bermudensis* sp. nov. anterior part, triple staining, dorso-lateral view, CLSM (NHMD-90364). E. Close up of *M. bermudensis* sp. nov. anterior part, single staining, Acetylated-α-tub-LIR (blue), dorsal view, CLSM (NHMD-90356). Abbreviations: asvn; anterior somata of ventral nerve net; cb1, cb2, 1. & 2. Ciliary bands; co, collar region; es, esophagus; hg, hindgut; mg, midgut; msvn, median somata of ventral nerve net; nc, neurochord (collar); pb, proboscis; pc, protocoel; plm, proboscis longitudinal muscles; pln, nerves innervating proboscis longitudinal muscles; psvn, posterior somata of ventral nerve net; scb2, somata of second ciliary band nerves; spb, somata of proboscis; snc, posterior somata of neurochord; tg, tail glands.

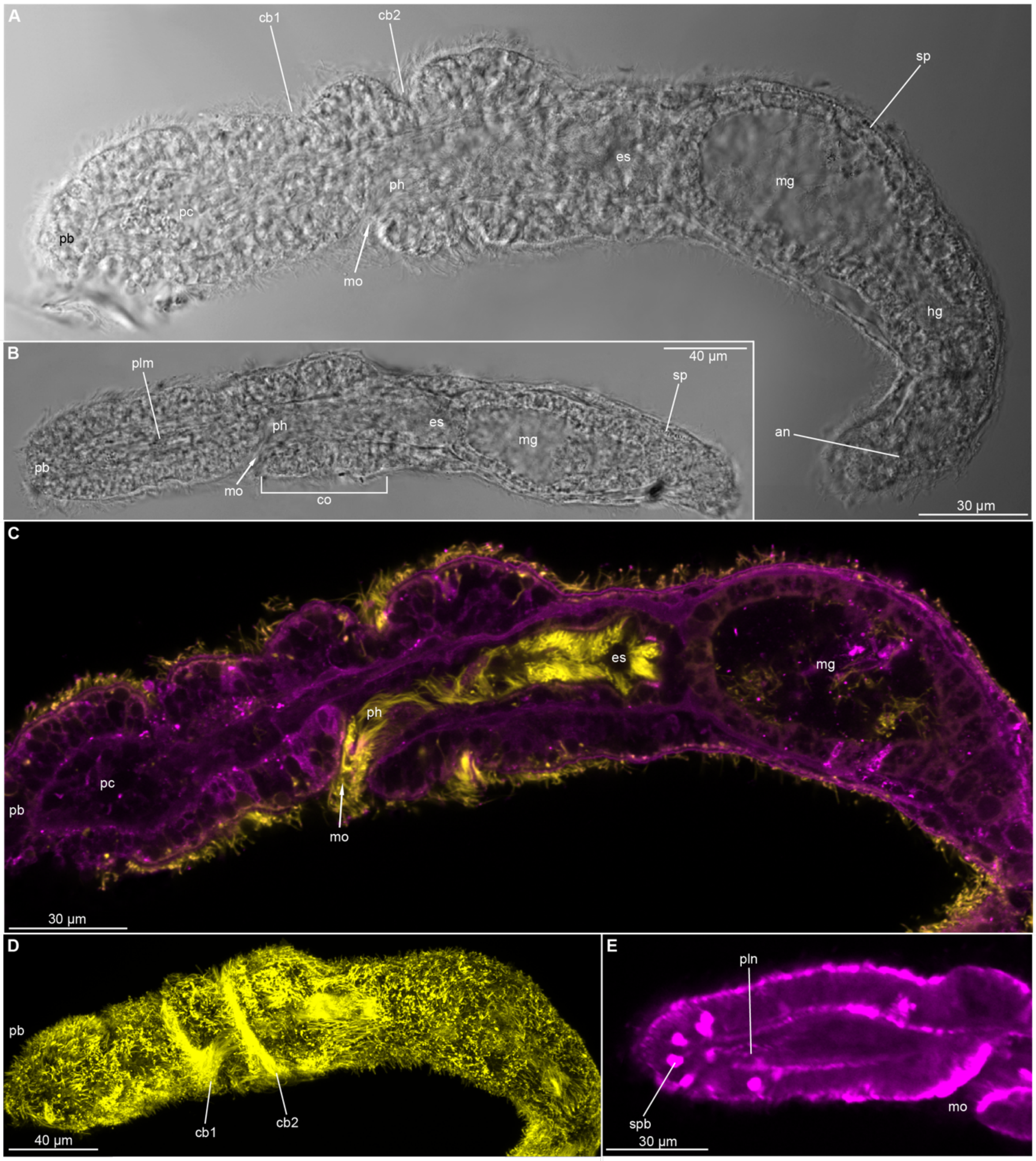

Supplementary Figure 6. *Meioglossus chiviricoensis* sp. nov., light- and confocal microscopy (LM and CLSM). A. Overview of *M. chiviricoensis* sp. nov., lateral view, LM (NHMD-1731259). B. Overview of *M. chiviricoensis* sp. nov., lateral view, LM (NHMD-1731260). C. Whole specimen of *M. chiviricoensis* sp. nov., double staining, Acetylated-α-tub-LIR (yellow) and FMRF-LIR (magenta), lateral view, CLSM (NHMD-1731259). D. Whole specimen of *M. chiviricoensis* sp. nov., single staining, Acetylated-α-tub-LIR (yellow), lateral view, CLSM (NHMD-1731259). E. Anterior part of specimen B., single staining, FMRF-LIR (magenta), lateral view, CLSM (NHMD-1731260). Abbreviations: an, anus; cb1, cb2, 1. & 2. Ciliary bands; co, collar region; es, esophagus; hg, hindgut; mg, midgut; mo, mouth opening; pb, proboscis; pc, protocoel; ph, pharynx; plm, proboscis longitudinal muscles; pln, nerves innervating proboscis longitudinal muscles; sp, sperm; spb, somata of proboscis.

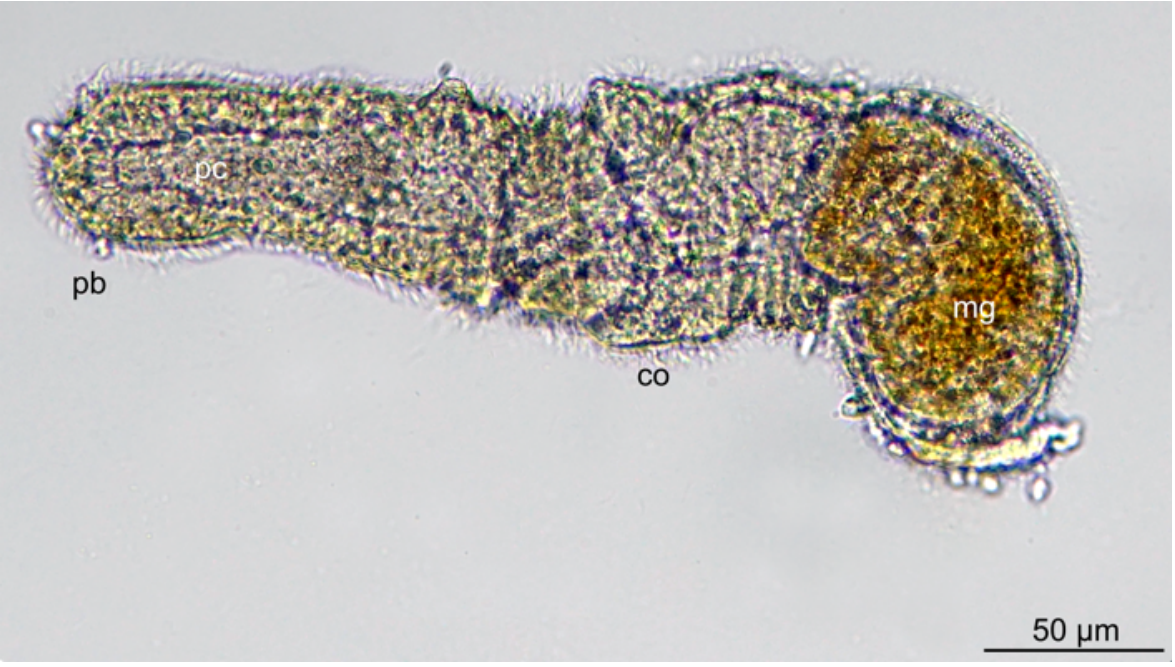

Supplementary Figure 7. Overview of *Meioglossus curacaoensis* sp. nov., lateral view, LM.

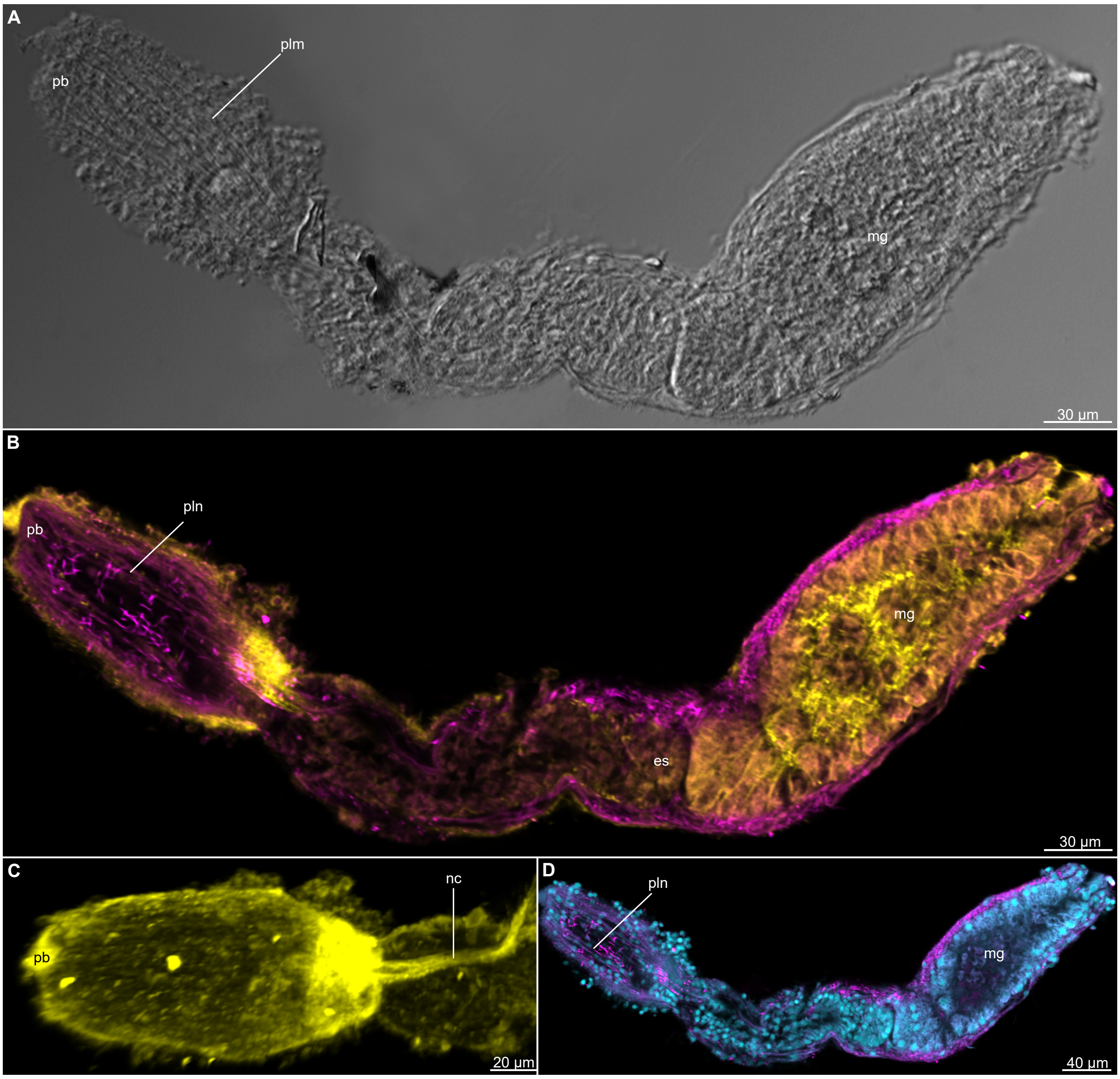

Supplementary Figure 8. *Meioglossus iuiensis* sp. nov., light- and confocal microscopy (LM and CLSM) (NHMD-1731267). A. Overview of *M. iuiensis* sp. nov., lateral view, LM. B. Whole specimen of *M. iuiensis* sp. nov., double staining, Acetylated-tyr-tub-LIR (yellow) and Serotonin-LIR (magenta), lateral view, CLSM. C. Anterior part of specimen B., single staining, Acetylated-tyr-tub-LIR (yellow), lateral view, CLSM. D. Whole specimen of *M. iuiensis* sp. nov., double staining, Serotonin-LIR (magenta) and DAPI (cyan), lateral view, CLSM. Abbreviations: es, esophagus; mg, midgut; nc, neurochord (collar); pb, proboscis; plm, proboscis longitudinal muscles; pln, nerves innervating proboscis longitudinal muscles.

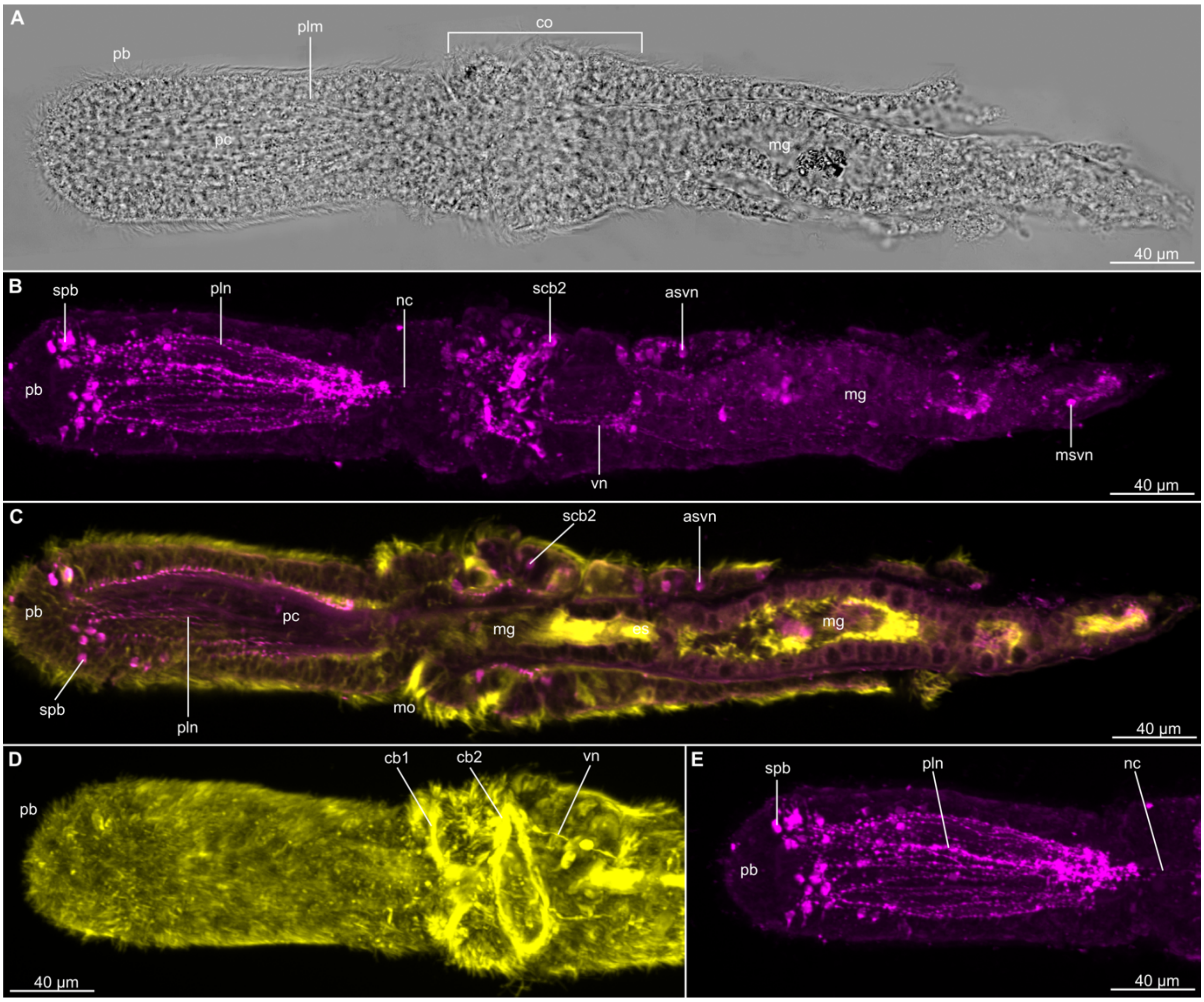

Supplementary Figure 9. *Meioglossus jejuensis* sp. nov., light- and confocal microscopy (LM and CLSM) (NHMD-1731269). A. Overview of *M. jejuensis* sp. nov., lateral view, LM. B. Whole specimen of *M. jejuensis* sp. nov., single staining, Serotonin-LIR (magenta), lateral view, CLSM. C. Whole specimen of *M. jejuensis* sp. nov., double staining, Acetylated-tyr-tub-LIR (yellow) and Serotonin-LIR (magenta), lateral view, CLSM. D. Anterior part of specimen B., single staining, Acetylated-tyr-tub-LIR (yellow), lateral view, CLSM. E. Anterior part of specimen B., single staining, Serotonin-LIR (magenta), lateral view, CLSM. Abbreviations: asvn; anterior somata of ventral nerve net; cb1, cb2, 1. & 2. ciliary bands; co, collar region; es, esophagus; mg, midgut; msvn, median somata of ventral nerve net; nc, neurochord (collar); pb, proboscis; pc, protocoel; plm, proboscis longitudinal muscles; pln, nerves innervating proboscis longitudinal muscles; scb2, somata of second ciliary band nerves; spb, somata of proboscis; snc, posterior somata of neurochord; vn, ventral nerve net (forms two ventral 'cords').

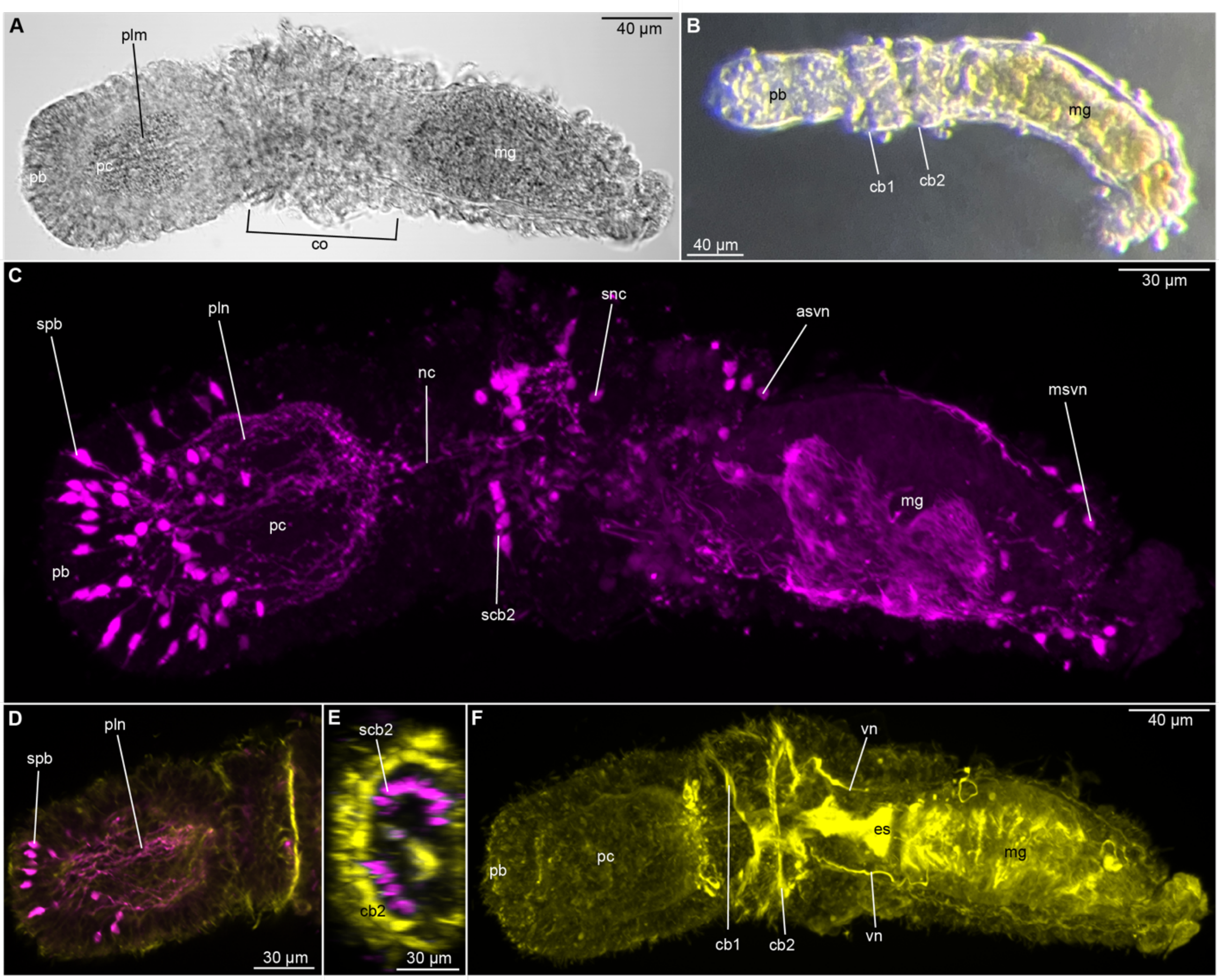

Supplementary Figure 10. *Meioglossus maldivensis* sp. nov., light- and confocal microscopy (LM and CLSM). A. Overview of *M. maldivensis* sp. nov., dorso-lateral view, LM (NHMD-1731275). B. Overview of *M. maldivensis* sp. nov., lateral view, dissecting scope. C. Whole specimen of *M. maldivensis* sp. nov., single staining, Serotonin-LIR (magenta), dorso-lateral view, CLSM (NHMD-1731275). D. Anterior part of specimen C., double staining, Acetylated-tyr-tub-LIR (yellow) and Serotonin-LIR (magenta), dorso-lateral view, CLSM (NHMD-1731275). E. Section 2. ciliary band of specimen C., double staining, Acetylated-tyr-tub-LIR (yellow) and Serotonin-LIR (magenta), CLSM (NHMD-1731275). F. Whole specimen of *M. maldivensis* sp. nov., single staining, Acetylated-tyr-tub-LIR (yellow), dorso-lateral view, CLSM (NHMD-1731275). Abbreviations: asvn; anterior somata of ventral nerve net; cb1, cb2, 1. & 2. ciliary bands; co, collar region; es, esophagus; mg, midgut; msvn, median somata of ventral nerve net; nc, neurochord (collar); pb, proboscis; pc, protocoel; plm, proboscis longitudinal muscles; pln, nerves innervating proboscis longitudinal muscles; psvn, posterior somata of ventral nerve net; scb2, somata of second ciliary band nerves; spb, somata of proboscis; snc, posterior somata of neurochord; vn, ventral nerve net (forms two ventral ‘cords’).

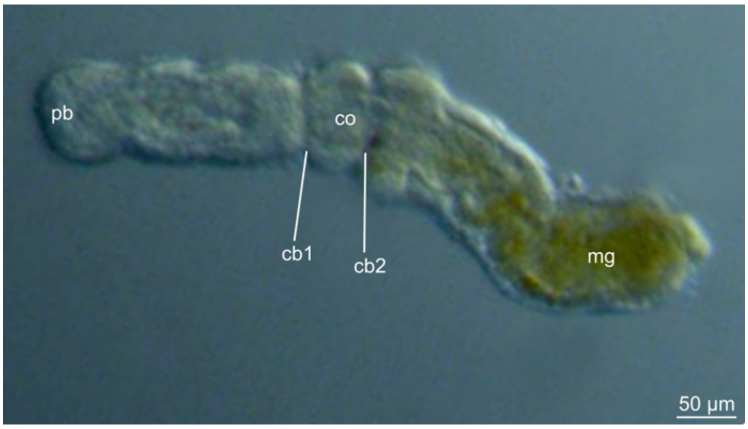

Supplementary Figure 11. Overview of *Meioglossus turkensis* sp. nov., lateral view, dissecting scope.

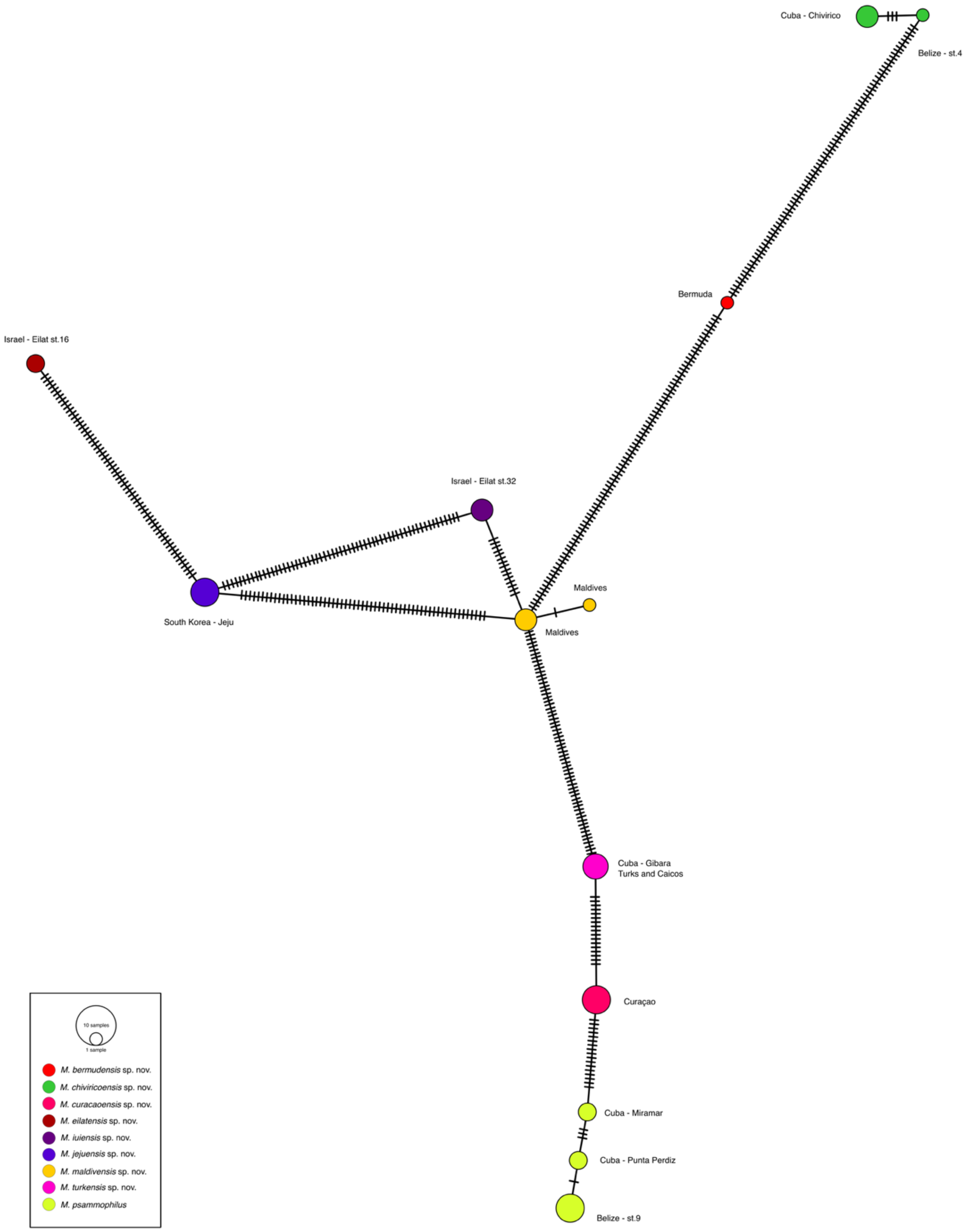

Supplementary Figure 12. Haplotype network using the 16S rRNA dataset.

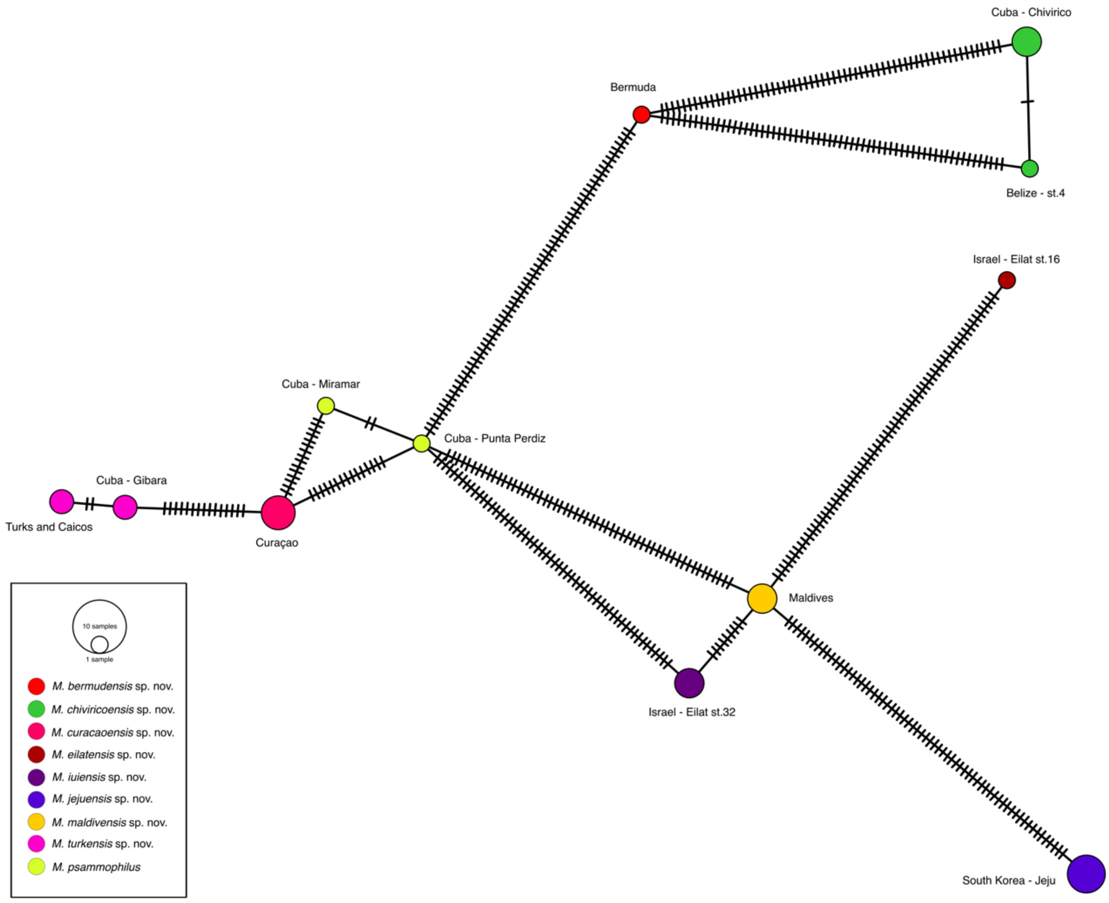

Supplementary Figure 13. Haplotype network using the COI dataset.

References

1. Palumbi, S. R. The polymerase chain reaction. *Molecular systematics*, 205-247. (1996).
2. Rousset, V., Pleijel, F., Rouse, G. W., Erséus, C., & Siddall, M. E. A molecular phylogeny of annelids. *Cladistics*. **23**(1), 41-63. https://doi.org/10.1111/j.1096-0031.2006.00128.x (2007).
3. Hillis, D. M., & Dixon, M. T. Ribosomal DNA: molecular evolution and phylogenetic inference. *Q. Rev. Biol.* **66**(4), 411-453. https://doi.org/10.1086/417338 (1991).
4. Whiting, M. F., Carpenter, J. C., Wheeler, Q. D., & Wheeler, W. C. The Strepsiptera problem: phylogeny of the holometabolous insect orders inferred from 18S and 28S ribosomal DNA sequences and morphology. *Syst. Biol.* **46**(1), 1-68. https://doi.org/10.1093/sysbio/46.1.1 (1997).
5. Giribet, G., Carranza, S., Baguña, J., Riutort, M., & Ribera, C. First molecular evidence for the existence of a Tardigrada + Arthropoda clade. *Mol. Biol. Evol.* **13**(1), 76-84. https://doi.org/10.1093/oxfordjournals.molbev.a025573 (1996).
6. Cohen, B. L., Améziane, N., Eleaume, M., & de Forges, B. R. Crinoid phylogeny: a preliminary analysis (Echinodermata: Crinoidea). *Mar. Biol.* **144**, 605-617. https://doi.org/10.1007/s00227-003-1212-7 (2004).
7. Lovejoy, C., & Potvin, M. Microbial eukaryotic distribution in a dynamic Beaufort Sea and the Arctic Ocean. *J. Plankton Res.* **33**(3), 431-444. https://doi.org/10.1093/plankt/fbq124 (2011).
8. Colgan, D. J., Hutchings, P. A., & Brown, S. Phylogenetic relationships within the Terebellomorpha. *J. Mar. Biolog. Assoc. U.K.* **81**(5), 765-773. https://doi.org/10.1017/S002531540100457X (2001).
9. Colgan, D. J., McLauchlan, A., Wilson, G. D., Livingston, S. P., Edgecombe, G. D., Macaranas, J., Cassis, G., & Gray, M. R. Histone H3 and U2 snRNA DNA sequences and arthropod molecular evolution. *Aust. J. Zool.* **46**(5), 419-437. https://doi.org/10.1071/ZO98048 (1998).
10. Worsaae, K., Sterrer, W., Kaul-Strehlow, S., Hay-Schmidt, A., & Giribet, G. An anatomical description of a miniaturized acorn worm (Hemichordata, Enteropneusta) with asexual reproduction by paratomy. *PLoS One*. **7**(11), e48529. https://doi.org/10.1371/journal.pone.0048529 (2012).
